# CX3CL1 Worsens Cardiorenal Dysfunction and Serves as a Therapeutic Target of Canagliflozin for Cardiorenal Syndrome

**DOI:** 10.3389/fphar.2022.848310

**Published:** 2022-03-18

**Authors:** Cankun Zheng, Wanling Xuan, Zhenhuan Chen, Rui Zhang, Xiaoxia Huang, Yingqi Zhu, Siyuan Ma, Kaitong Chen, Lu Chen, Mingyuan He, Hairuo Lin, Wangjun Liao, Jianping Bin, Yulin Liao

**Affiliations:** ^1^Department of Cardiology, State Key Laboratory of Organ Failure Research, Guangdong Provincial Key Laboratory of Shock and Microcirculation, Nanfang Hospital, Southern Medical University, Guangzhou, China; ^2^Department of Cardiology, Jiangxi Provincial People’s Hospital Affiliated to Nanchang University, Nanchang, China; ^3^Department of Oncology, Nanfang Hospital, Southern Medical University, Guangzhou, China; ^4^National Clinical Research Center of Kidney Disease, Guangdong Provincial Institute of Nephrology, Nanfang Hospital, Southern Medical University, Guangzhou, China

**Keywords:** cardiorenal syndrome, CX3CL1, apoptosis, cellular transition, sodium glucose cotransporter 2 inhibitor

## Abstract

The prognosis of cardiorenal dysfunction induced by diabetes mellitus (DM), which belongs to cardiorenal syndrome type 5, is poor and its pathogenesis remains elusive. We have reported that CX3CL1 exacerbated heart failure and direct inhibition of CX3CL1 improved cardiac function. Emerging evidence supports that CX3CL1 is involved in renal impairment. Here we attempt to clarify whether CX3CL1 might be a therapeutic target for cardiorenal dysfunction in diabetes. We found that cardiac and renal CX3CL1 protein levels were significantly increased in both streptozotocin-induced diabetic mice and in non-obese diabetic mice, and that hyperglycemia led to persistent CX3CL1 expression in the heart and kidneys even after it was controlled by insulin. In cultured cardiac and renal cells, soluble CX3CL1 accelerated mitochondrial-dependent apoptosis via activation of the RhoA/ROCK1-Bax signaling pathway and promoted fibrosis through cellular phenotypic trans-differentiation mediated by the TGF-β/Smad pathway. In the two diabetic mouse models, knockout of CX3CL1 receptor CX3CR1 or treatment with an CX3CL1 neutralizing antibody significantly improved cardiorenal dysfunction by inhibiting apoptosis, mitochondrial dysfunction, and fibrosis. Moreover, sodium glucose cotransporter 2 inhibitor canagliflozin significantly downregulated cardiac and renal CX3CL1 expression and improved cardiorenal dysfunction. These findings indicate that CX3CL1 could be a new therapeutic target for diabetes-induced cardiorenal dysfunction.

## Introduction

The prevalence and mortality of diabetes mellitus (DM) are increasing rapidly worldwide, and the majority of diabetic patients ultimately succumb to multiple complications, so the coexistence of cardiac and renal dysfunction in these patients is frequent. The cardiorenal syndrome (CRS) associated with DM is a systemic condition that leads to abnormalities of both the cardiovascular and renal systems (CRS type 5) (Karnib and Ziyadeh, 2010). The prognosis of diabetic patients with CRS is poor. To date, strict glycemic control is still the principal therapeutic approach, but there is evidence from laboratory studies and from large-scale clinical trials in patients with type 1 and type 2 DM that diabetic complications continue to progress through the phenomenon of metabolic memory even after glycemic control has been achieved (Ceriello, 2012). The early glycemic environment is imprinted on target organs (including the eyes, kidneys, heart, and extremities), and this phenomenon has been termed “metabolic memory” (Ihnat et al., 2007). The mechanisms of this “memory” are not fully understood, but have been suggested to include the overproduction of reactive oxygen species unrelated to the presence of hyperglycemia, production of advanced glycation end products, or alterations of gene expression induced by epigenetic mechanisms that persist after the glucose level is normalized (Ceriello, 2012; Saremi et al., 2017; Natarajan, 2021; Yao et al., 2021).

CX3CL1 is a chemokine that acts as a chemoattractant and an adhesion molecule via its specific receptor (CX3CR1) (Umehara et al., 2004). Elevated circulating levels of CX3CL1 were detected in patients with type 2 DM, suggesting a link between CX3CL1 and diabetes (Shah et al., 2011). In addition, we have demonstrated that CX3CL1 exacerbates heart failure and promotes cardiac remodeling in mice with myocardial ischemia or pressure overload (Xuan et al., 2011). Moreover, there is emerging evidence that CX3CL1 may also be involved in renal failure and diabetic nephropathy (Oh et al., 2008; Koziolek et al., 2010; Song et al., 2013; Von Vietinghoff and Kurts, 2021; Woźny et al., 2021). However, the influence of the CX3CL1/CX3CR1 system on CRS caused by DM remains unclear.

Even the goal of strict glycemic control was reached, some glucose-lowering agents such as rosiglitazone and saxagliptin were observed to increase the risk of heart failure (HF) (Savarese et al., 2021), while glucagon-like peptide-1 receptor agonists and sodium-glucose cotransporter-2 inhibitors (SGLT2i) have been demonstrated to be protective for cardiorenal dysfunction (Brown et al., 2021; Li et al., 2021). These findings indicate the importance of cardiovascular outcome in the use of antidiabetic agents and optimal antidiabetic treatments are likely to attenuate the damage of metabolism memory. It was reported that chronic CX3CL1 administration improves glucose tolerance in high-fat diet mice (Lee et al., 2013; Riopel et al., 2018), suggesting CX3CL1 is beneficial for DM treatment, but it is too early to say that CX3CL1 can be used to treat patients with DM before clarifying the cardiovascular outcome of CX3CL1. We hypothesized that CX3CL1 would promote cardiorenal dysfunction in DM, and inhibition of CX3CL1 would be a new therapeutic target for improving DM-induced cardiorenal syndrome.

In this study, we investigated the potential role of CX3CL1 in cardiorenal dysfunction by using streptozotocin (STZ)-induced DM or spontaneously diabetic mice (non-obese diabetes, NOD). We found that soluble CX3CL1 (sCX3CL1) promoted cardiorenal dysfunction in diabetic mice through proapoptotic and profibrotic effects. Moreover, canagliflozin could inhibit hyperglycemia-induced CX3CL1 expression in cardiac and renal cells and improved cardiorenal dysfunction in diabetic mice. Our findings suggest a new therapeutic target to alleviate cardiorenal dysfunction of CRS5 by inhibiting the CX3CL1/CX3CR1 system.

## Materials and Methods

All procedures were performed in accordance with our Institutional Guidelines for Animal Research and complied with the Guide for the Care and Use of Laboratory Animals published by the United States National Institutes of Health. The detailed methods are shown in the Supplementary Material online.

### STZ-Induced Diabetic Mice

Ten-week-old male C57BL/6 mice (wildtype, WT) or CX3CR1^−/−^ mice purchased from Jackson Laboratory were injected intraperitoneally with a single dose of STZ (Sigma Chemicals, United States) at 150 mg/kg dissolved in 10 mmol/L sodium citrate buffer (pH 4.5), while control mice were injected with the buffer alone. Three days later, mice with a random blood glucose level >20 mmol/L were assigned to the hyperglycemic groups, whereas citrate buffer-treated mice were assigned to the normoglycemic groups. The following experimental groups were formed: (1) WT mice (WT&Vehicle); (2) CX3CR1^−/−^ mice (CX3CR1^−/−^&Vehicle); (3) WT mice with STZ-induced DM (WT&STZ); (4) CX3CR1^−/−^ mice with STZ-induced DM (CX3CR1^−/−^&STZ); (5) WT mice with DM and insulin treatment for the final 2 weeks of the study (WT&STZ&Insulin), and (6) WT mice with DM and CX3CL1 neutralizing antibody treatment for the final 2 weeks of the study (WT&STZ&anti-CX3CL1). For insulin treatment, diabetic WT mice were injected subcutaneously with long-acting insulin at 40 IU/kg/day (Lantus; Sanofi-Aventis Deutschland GmbH, Germany). For treatment intervention, diabetic WT mice were injected intraperitoneally with anti-CX3CL1 antibody at 40 mg/kg/day (TP233; Torrey Pines Biolabs, Houston, TX, United States) as described previously (Xuan et al., 2011) or oral administration of canagliflozin at 10 mg/kg/day (Selleck Chemicals, Germany). Mice were euthanized by an overdose of pentobarbital (150 mg/kg, i.p) at 6 weeks after STZ injection to induce DM, and cardiac and renal function, apoptosis, and fibrosis were analyzed.

### NOD Mice

Female NOD mice, 10–30 weeks of age, were monitored for diabetes onset twice time per week by measurement of blood glucose. The mice with twice random blood glucose level >20 mmol/L were assigned to the hyperglycemic groups (day 1 of study). At 30^th^ week, the NOD mice with normal glucose were used as controls (NG). The treatment with CX3CL1 neutralizing antibody and insulin treatment in NOD mice was the same as in STZ mice mentioned above. Under anaesthetized by intraperitoneal injection of a mixture of xylazine (5 mg/kg) and ketamine (100 mg/kg), some NOD mice were injected with adeno-associated virus serotype 9 (AAV9) carrying the short hairpin-CX3CR1 plasmid (sh-CX3CR1) or a scramble plasmid (sh-scr) into the myocardium or kidneys. NOD mice were euthanized by an overdose of pentobarbital (150 mg/kg, i.p) and tissues harvest were performed for further analysis.

### Cell Culture

The neonatal rats were sacrificed by 2% isoflurane inhalation and cervical dislocation. Isolation and culture of ventricular cardiomyocytes (NRCM) and cardiac fibroblasts (NRCF) were performed as described previously with modifications (Korhonen et al., 2009; Tiede et al., 2010). After digesting the ventricular tissue, the cells were pelleted and the cells were re-suspended in Dulbecco’s modified Eagle’s medium (DMEM) containing 10% fetal bovine serum (FBS) and 1% penicillin-streptomycin. The resulting cell mixture was pre-plated for 1 h to plate out cardiac fibroblasts. The isolated cardiomyocytes were plated into 6-well plates and cultured for 4–5 days. Pre-plated cardiac fibroblasts were cultured for 2 days and then 2-4 cells were used to passage.

A rat proximal tubular cell line, NRK-52E (ATCC, United States), and a rat glomerular mesangial cell line, HBZY-1 (ATCC, United States) were maintained in Dulbecco’s modified Eagle’s medium (DMEM, Gibco) containing 10% FBS at 37°C.

Cultured NRCM and HBZY-1 were starved of serum for 12 h and then were exposed to recombinant soluble CX3CL1 (chemokine domain; 537-FT-025; R&D) at 200 ng/ml for 24 h in the presence or absence of 10 μM Y27632 (a ROCK inhibitor; Selleck) or 5 μg/ml of an anti-CX3CR1 antibody (GTX27200; Genetex). Cultured NRCF and NRK-52E were also exposed to recombinant soluble CX3CL1 (chemokine domain) at 200 ng/ml for 24 h in the presence or absence of 500 nM LY2157299 (a TGF-β/Smad signaling inhibitor; Selleck) or the anti-CX3CR1 antibody (5 μg/ml). Cultured H9C2, HBZY-1, NRCF, and NRK-52E were respectively exposed for 5 days either to normal glucose (5 mM, NG) or high glucose concentration (33.3 mM, HG) as well as to high glucose for 3 days followed by normal glucose for the remaining 2 days (HN) with/without different dose of canagliflozin (1, 5, and 10 μM).

### RhoA Assay

RhoA GTPase activity was measured in cell lysates prepared from cardiomyocytes or glomerular mesangial cells in the presence or absence of 200 ng/ml sCX3CL1 by using a pull-down assay that employed the Rho-binding domain of the Rho effector protein rhotekin (Cytoskeleton, Inc., Denver, CO, United States, BK036). Total cell extracts and precipitates were analyzed by sodium dodecyl sulfate-polyacrylamide gel electrophoresis (SDS-PAGE) and Western blotting with a monoclonal antibody targeting RhoA (diluted 1:500).

### Cellular Distribution of Cytochrome C and Apoptosis-Inducing Factor

Confocal microscopy was used to delineate the distribution of cytochrome C. Cells were incubated with 25 nM of Mitotracker dye (Mitotracker red; Molecular Probes, Invitrogen) at 37°C for 10 min. Slides were washed with PBS and fixed in 4% formaldehyde. They were then incubated with primary anti-cytochrome C or AIF antibody followed by incubation with Alex488-labeled secondary antibody. The cells were also subjected to DAPI staining to visualize the nuclei. Images were taken using different excitation filters and merged.

### Immunofluorescence Staining

For immunofluorescence staining, cultured cells were fixed in 3.7% paraformaldehyde for 10 min and then permeabilized with 0.1% Triton X-100 for 5 min. Next, the cells were incubated for 30 min at room temperature with blocking buffer (PBS containing 20% goat serum), before being incubated with the primary antibodies overnight at 4°C. The negative control was incubated with PBS instead of the primary antibody. For staining of heart and kidney sections, tissues were first embedded in Tissue-Tech OCT compound in cryomolds and then were snap-frozen in 2-methlybutane pre-cooled in liquid nitrogen and stored at −80°C. Heart and kidney sections were cut at a thickness of 5 μm and fixed for 10 min with chilled acetone. After blocking with 10% normal donkey serum in PBS for 30 min, sections were incubated with the primary antibodies overnight at 4°C. Next, the corresponding secondary antibodies conjugated to Alex Fluor 488 dyes (Invitrogen, United States) or Cy3 dyes were added and the sections were incubated for 60 min at room temperature. Nuclei were counterstained with DAPI. Images were acquired with a confocal laser microscope (Olympus, Japan).

### Immunohistochemistry

Heart and kidney tissues from the different groups mice were fixed in 4% paraformaldehyde and embedded in paraffin. Then 4 μm sections were prepared for immunostaining. After antigen retrieved by heating in citrate buffer (pH 6.0), the sections were incubated with goat anti-CX3CL1 and anti-Bax antibodies, followed by incubation with an HRP-labeled secondary antibody. The Dako EVision + System-HRP (DAB) was used to visualize CX3CL1 and Bax staining. Images were captured using an upright microscope (Olympus, Japan).

### Calcein-AM Assay and Measurement of the Mitochondrial Membrane Potential (Δψm)

After sCX3CL1 treatment for 24 h, cardiomyocytes or glomerular mesangial cells were loaded with 2 μM calcein-AM (Molecular Probes, United States) and 5 mM cobalt chloride for 15 min at 37°C to quench cytosolic calcein fluorescence (Dai et al., 2011). Then the cells were rinsed to remove residual dye and opening of the mPTP was visualized as redistribution of calcein from the mitochondrial matrix to the cytosol. For assessment of the mitochondrial membrane potential, cells were stimulated with sCX3CL1 for 24 h, loaded with 50 nmol/L tetramethylrhodamine ethyl (TMRE) for 30 min at 37°C, and then stained with DAPI for 10 min at room temperature. Viable cells were observed through a confocal laser microscope (Olympus, Japan). The fluorescence intensity of calcein-AM was monitored at 515 nm, while the fluorescence intensity of TMRE was monitored at 582 nm. A total of 200 cells from two replicate dishes were assessed for fluorescence intensity in three independent experiments, with the relative fluorescence intensity being determined by Image-Pro Plus 6.0 software (United States).

### TUNEL Staining

To localize nuclear DNA fragmentation in cultured cells or heart and kidney tissues, *in situ* terminal deoxynucleotidyl transferase-mediated dUTP nick-end labeling (TUNEL) was performed using commercial apoptosis detection kit (Merck, United States or Roche, United States). All procedures were done according to the directions of the manufacturers. Cells were counterstained with DAPI to visualize nuclei. The number of TUNEL-positive cells was determined by randomly counting 10 fields of a section and was expressed as a percentage of the cells with normal nuclei.

### Transmission Electron Microscopy

Heart and kidney tissues were processed for electron microscopy as described previously to assess mitochondrial changes in the myocardium and renal tubules (Bernardi et al., 1999).

Tissues were cut into pieces smaller than 1 mm3 and fixed in 2.5% glutaraldehyde in 0.1 M sodium cacodylate buffer (pH 7.4) for 4 h. Ten the tissues were post-fixed in 1% osmium tetroxide in 1% K4Fe(CN)6 buffer with 0.1 M sodium cacodylate, dehydrated through a graded series of ethanol and propylene oxide, and embedded in Epon 812. Ultrathin sections were cut, mounted on copper grids, and stained with lead citrate and uranyl acetate. A Hitachi H-7600 transmission electron microscope (Pleasanton, CA, United States) equipped with a digital camera was used to capture images of the sections. Damaged mitochondria were defined as those showing vacuolation, disruption of cristae, or deformity.

### Nuclear DNA Fragmentation Assay

Nuclear DNA fragmentation was assessed by gel electrophoresis using the apoptotic DNA-ladder kit (Roche, United States) according to the manufacturer’s instructions. Briefly, after DNA extraction, sample DNA concentrations were quantified by reading the relative absorbance at 260/280, and 40 mg of DNA from each sample was loaded onto 1% agarose gel, separated by electrophoresis, stained by ethidium bromide, and visualized with an ultraviolet trans-illuminator.

### Masson’s Trichrome and Morphometric Analysis

Paraffin-embedded left ventricular sections were stained with Masson’s trichrome to measure interstitial fibrosis with the Image-Pro Plus 6.0 software. Myocardial interstitial fibrosis and perivascular fibrosis were quantified using a modification of the technique described (Liao et al., 2003; Ti et al., 2011). Briefly, ten 400x fields from 10 random stained sections from the mid left ventricle were digitized. For myocardial interstitial fibrosis analysis, an area stained blue was then selected for its color range, and the proportional area stained blue (matrix) was then determined. Perivascular fibrosis was excluded from the interstitial fibrosis measurement. For perivascular fibrosis measurement, perivascular fibrosis area/luminal area (PVCA/LA) was analyzed. To normalize the PVCA around vessels with different sizes, the perivascular fibrosis was represented as the PVCA-to-LA ratio. Kidney sections were stained with Masson’s trichrome. For the semiquantitative evaluation of fibrosis by Masson-Trichrome staining in the kidney, renal fibrotic lesions were expressed as percentage of Masson’s trichrome-positive fibrotic area. 20 randomly selected glomerulus or tubulointerstitial areas per mouse were graded as reported previously (Kitada et al., 2011).

### Renal Function Assessment

After 6 weeks of DM induction, individual mice were placed in metabolic cages for 24 h urine collection. The urine samples were stored at -80°C until analysis. Blood samples were collected from the left cardiac ventricle. Enzyme-Linked Immunosorbent Assay (ELISA) was used to measure the level of neutrophil gelatinase-associated lipocalin (NGAL) in serum or 10% supernatant fluid of kidney tissues, serum creatinine (Cr), urine Cr, and serum blood urea nitrogen (BUN) from mice by using the kits (cusabio, United States) according to the protocols of the manufacturers.

### Echocardiographic Analysis

Cardiac function was evaluated in mice by echocardiography using a Sequoia 512 system with a 15L-8 probe (Siemens, Germany). Mice were anaesthetized with inhalational isoflurane at a concentration of 1.5%, and two-dimensional parasternal short-axis images of the left ventricle (LV) were obtained at the level of the papillary muscles. From M-mode tracings, the LV end-diastolic diameter (LVEDD), LV end-systolic diameter (LVESD), and LV fractional shortening (LVFS) were measured.

### RNA Isolation and PCR

Total RNA was extracted from culture cells and mouse heart and kidney tissues (total RNA isolation system, Omega, United States). Reverse transcription was carried out in 20 μL of reaction mixture containing 1 μg of total RNA. Digital images of CX3CL1, CX3CR1, and β-actin bands separated on ethidium bromide-stained agarose gels were obtained. Primer sequence was listed in the Supplementary Table S1.

### Western Blot Analysis

Samples containing equal amounts of protein were separated by 8–12% sodium dodecyl sulfate-polyacrylamide gel electrophoresis and transferred onto polyvinyl difluoride membranes. The membranes were blocked with 5% skim milk at room temperature for 1 h, incubated overnight at 4°C with the primary antibodies (Supplementary Table S2), and then washed three times with TBST buffer before incubation for 1 h with HRP-conjugated secondary antibodies at room temperature. The blots were detected using with Dylight 800-labeled secondary antibodies in a Western blotting detection system (Infrared Imaging System, Licor, United States). Relative expression was quantified by densitometry using the ImageJ Analysis Software (National Institutes of Health, Bethesda, MD, United States).

### Statistical Analysis

Data are expressed as the mean ± SEM. Comparison of two groups was performed by Student’s *t*-test, while multiple comparisons were done by one-way ANOVA followed by Bonferroni’s multiple comparison exact probability test. All analyses were performed with GraphPad Prism 8.0 software (GraphPad Software Inc., CA, United States), and Significance was accepted at *p* < 0.05.

## Results

### High Blood Glucose Upregulates CX3CL1 in Cardiac and Renal Tissues

**FIGURE 1:**
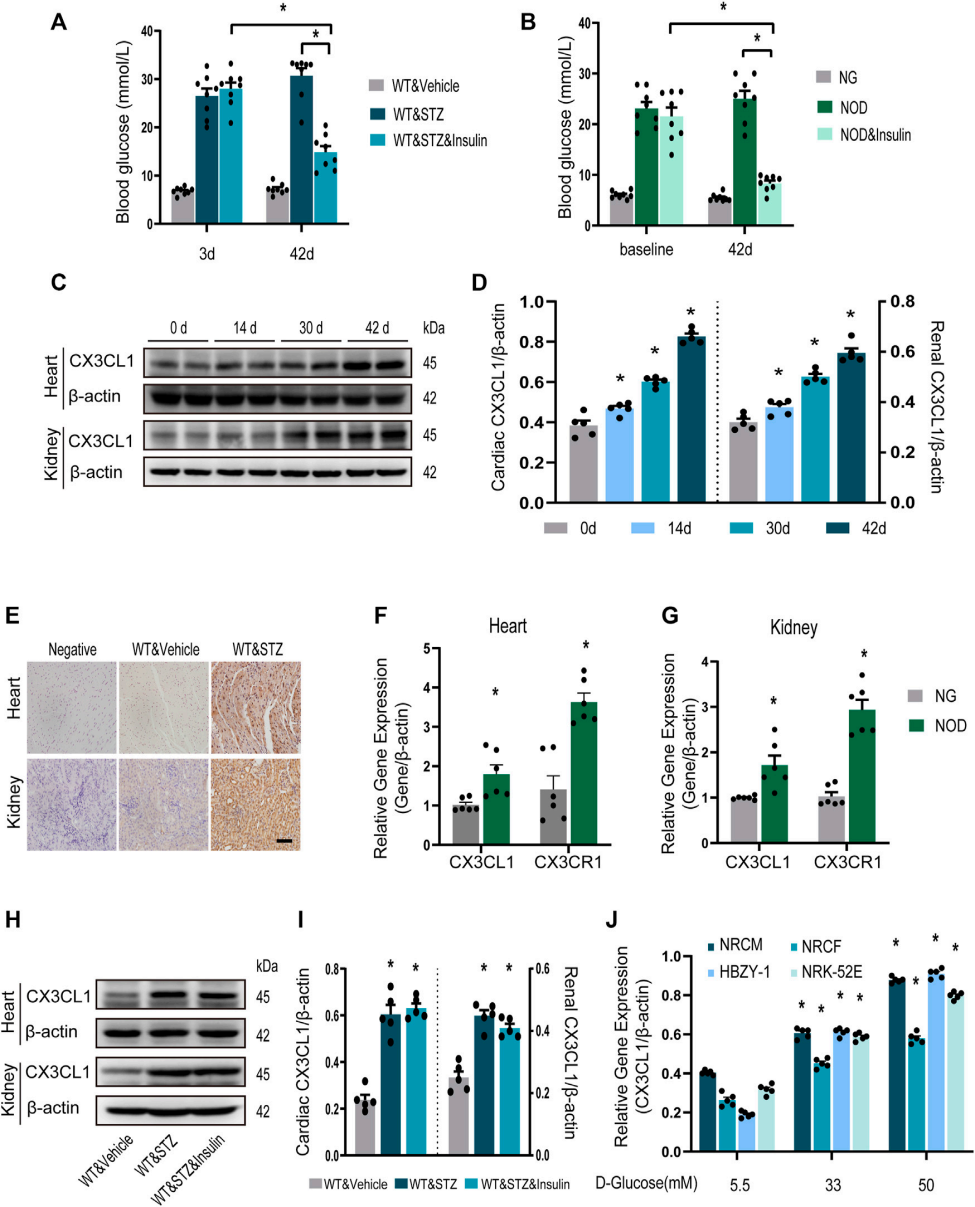
CX3CL1 expression in response to glucose stimulation. **(A)** Blood glucose levels at 3 and 42 days in streptozotocin (STZ)-induced DM mice. Insulin treatment was initiated at 28 days after STZ injection and persisted for 14 days. ^*^p < 0.05, *n* = 8 in each group. **(B)** Blood glucose levels at 3 and 42 days in NOD mice. The NOD mice were assigned to three groups: normal glucose (NG) group (some mice didn’t develop into hyperglycemia), NOD group (with twice random blood glucose level>20 mM) and NOD&insulin group. Insulin treatment protocol was the same as panel **(A)**. ^*^p < 0.05, *n* = 8 in each group. **(C)** Representative western blotting of CX3CL1 in the heart and kidney tissue of mice at various time points after STZ-injection. **(D)** Semi-quantitation of CX3CL1 expression in panel **(C)**. ^*^p < 0.05, *vs*. the corresponding 0 day, *n* = 5 per group. **(E)** Immunohistochemical detection of CX3CL1 expression in heart and kidney tissues of normal or diabetic mice. Scale bar = 100 μm. **(F)** Cardiac CX3CL1 and CX3CR1 mRNA expression in NOD mice. **(G)** Renal gene expression of CX3CL1 and CX3CR1 in NOD mice. ^*^p < 0.05, *vs*. NG, *n* = 6 per group. **(H)** CX3CL1 expression detected by western blotting in heart and kidney tissues from DM mice with or without insulin treatment for 14 days. **(I)** Semi-quantitation of CX3CL1 in panel **(H)**. ^*^p < 0.05 *vs*. WT&Vehicle, *n* = 5 in each group. **(J)** mRNA expression of CX3CL1 in neonatal rat cardiomyocytes (NRCM) and fibroblasts (NRCF), two types of renal cell lines HBZY-1 and NRK-52E exposed to different concentrations of glucose medium for 3 days. ^*^p < 0.05 *vs*. 5.5 mM group, *n* = 5 in each group. Experiments presented in panels **(A)**, **(B)**, **(D)**, **(I)**, and **(J)** were analyzed using one-way ANOVA followed by Bonferroni’s *post hoc* test. Experiments presented in panels **(F)** and **(G)** were analyzed using two-tailed unpaired *t*-test.

Three days after STZ injection in mice, blood glucose levels were increased by about six folds. And they were almost normalized by insulin treatment for the final 14 days of the study (Figure 1A). Similar results were obtained in NOD mice (Figure 1B). CX3CL1 protein expression in the heart and kidneys was significantly increased in a time-dependent manner throughout the 42-day study period (Figures 1C,D). Immunohistochemistry showed that CX3CL1 expression was dramatically enhanced in the heart and kidney tissues of mice with STZ injection (Figure 1E). The gene expression levels of CX3CL1 and its receptor CX3CR1 were also increased in hearts and kidneys of NOD mice (Figures 1F,G).

We then investigated whether enhanced CX3CL1 expression would persist after hyperglycemia was controlled. From 4 weeks after STZ injection, mice with DM were treated with insulin for 2 weeks. Western blotting revealed that myocardial and renal CX3CL1 protein expression was not significantly lower after the control of hyperglycemia than in the untreated group (Figures 1H,I), suggesting that upregulation of CX3CL1 may represent a molecular memory of hyperglycemia. Moreover, we measured CX3CL1 expression in the principal cell types of the heart or kidney, which were exposed to high glucose. Compared with exposure to 5.5 mM D-glucose for 72 h, exposure to high concentrations of D-glucose (33 and 50 mM) resulted in a concentration-dependent increase of CX3CL1 expression in NRCM, NRCF, HBZY-1, and NRK-52E cells (Figure 1J).

### SCX3CL1 Promotes Apoptosis Through Induction of Mitochondrial Dysfunction

**FIGURE 2:**
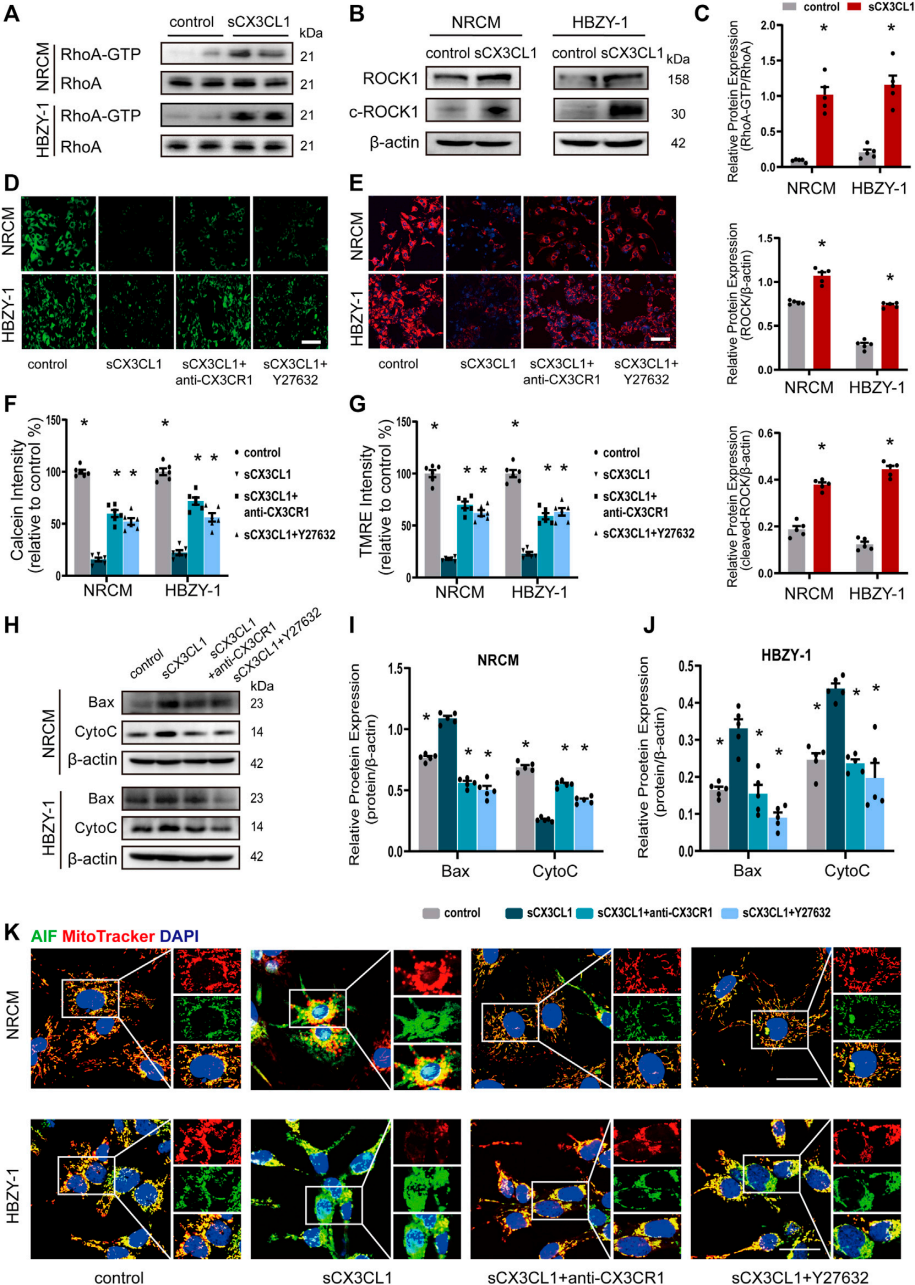
sCX3CL1 promotes mitochondrial-dependent apoptosis in cardiomyocytes and renal cells. **(A)** Western blotting to detect RhoA and GTP-bound RhoA protein in neonatal rat cardiomyocytes (NRCM) and HBZY-1 cells after 60 min of stimulation with 200 ng/ml of sCX3CL1 (soluble CX3CL1). **(B)** Western blotting to detect upregulation of ROCK1 and cleaved ROCK1 (c-ROCK) expression in response to stimulation of NRCM and HBZY-1 cells with sCX3CL1 (200 ng/ml) for 24 h. **(C)** Semi-quantitation of RhoA-GTP, ROCK and cleaved-ROCK expression. ^*^p < 0.05 *vs*. control, *n* = 5 in each group. Representative images of **(D)** calcein fluorescence or **(E)** TMRE fluorescence (Mitochondrial membrane potential) in cells cultured with sCX3CL1 alone or co-treated with either a CX3CR1 neutralizing antibody or a Rho kinase inhibitor (Y-27632). Scale bar = 100 μm. Semi-quantitative analysis of calcein **(F)** or TMRE **(G)** fluorescence intensity (^*^p < 0.05 *vs*. sCX3CL1), *n* = 5 in each group. **(H)** Western blots analysis of Bax, cytochrome C (Cyto C) in NRCM and HBZY-1. Semi-quantitation analysis of Bax and Cyto C in NRCM **(I)** and HBZY-1 cells **(J)**. ^*^p < 0.05 *vs*. sCX3CL1, *n* = 5 in each group. **(K)** Subcellular localization of AIF (apoptosis inducing factor) was detected in NRCM and HBZY-1 cells after sCX3CL1 stimulation or co-treatment with either a CX3CR1 neutralizing antibody or Y-27632. Scale bar = 30 μm. Experiments presented in panel **(C)** was analyzed using two-tailed unpaired *t*-test and in panels **(F)**, **(G)**, **(I)**, and **(J)** were analyzed using one-way ANOVA followed by Bonferroni’s *post hoc* test.

Treatment with soluble CX3CL1 (sCX3CL1) for 1 h directly activated RhoA GTPase in both NRCM and HBZY-1 cells (Figures 2A,C). The expression of intact and cleaved ROCK1 protein was significantly upregulated in NRCM and HBZY-1 cells after exposure to sCX3CL1 for 24 h (Figures 2B,C). There was a decrease of fluorescence intensity detected by the calcium plus CoCl_2_ assay in NRCM and HBZY-1 cells following sCX3CL1 stimulation (Figures 2D,F). Confocal microscopy revealed loss of TMRE staining, which indicates a decrease of the mitochondrial membrane potential, in cardiac and renal cells after stimulation with sCX3CL1 (Figures 2E,G). These findings indicate that the mitochondrial permeability transition was enhanced by sCX3CL1. In NRCM and HBZY-1 cells, stimulation with sCX3CL1 significantly increased mitochondrial-dependent apoptosis pathway related marker, include Bax and cytochrome C (CytoC) (Figures 2H–J). And after identifying mitochondria and nuclei by staining with Mitotracker (red) and DAPI (blue), respectively, the subcellular distribution of AIF and cytochrome C was monitored with confocal microscopy by detecting green immunofluorescence. This showed that sCX3CL1 stimulation induced translocation of AIF into the nucleus (Figure 2K) and the release of CytoC from the mitochondria into the cytoplasm (Supplementary Figures S1A,B). Moreover, the TUNEL and DNA ladder assays showed that sCX3CL1 increased apoptosis and DNA fragmentation in cultured cells (Supplementary Figures S1C–G). We also found that sCX3CL1 stimulated caspase-9 activity (Supplementary Figures S2A,B). The above effects of sCX3CL1 were antagonized by co-treatment with either a CX3CR1 neutralizing antibody (5 μg/ml) or a Rho kinase inhibitor (Y-27632 at 10 μM) (Figures 2A–K; Supplementary Figures S1A–G; Supplementary Figures S2A,B). Taken together, our findings indicate that sCX3CL1 promotes the mitochondrial apoptotic pathway through activation of RhoA/ROCK1-Bax signaling.

### SCX3CL1 Promotes Phenotypic Trans-Differentiation of Cardiac and Renal Cells

**FIGURE 3:**
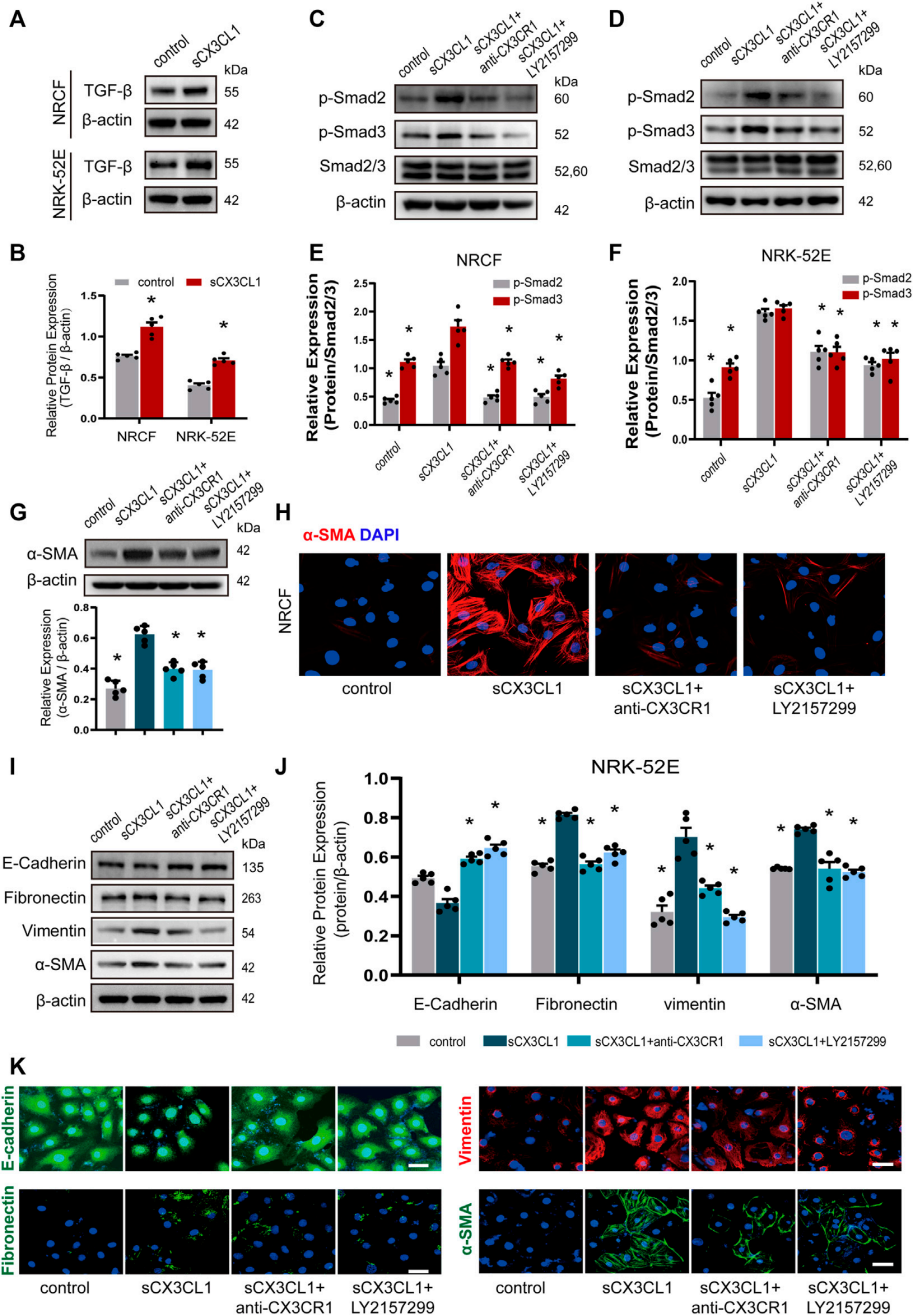
sCX3CL1 promotes cardiac and renal cells transformation in a TGF-β/Smad dependent manner. Representative western blots **(A)** and semi-quantitative analysis **(B)** of TGF-β expression in neonatal cardiac fibroblasts (NRCF) and a renal tubular epithelial cell line (NRK-52E) in response to sCX3CL1 stimulation for 24 h. Western blotting **(C)** and **(D)** and semi-quantitative analysis **(E)** and **(F)** of phosphorylation of Smad2 and Samd3 in NRCF and NRK-52E cells after exposure to sCX3CL1 stimulation alone or with co-treatment by either a CX3CR1 neutralizing antibody or a TGF-β/Smad inhibitor (LY2157299). **(G)** Marked upregulation of α-SMA protein detected by Western blotting in NRCF after stimulation with sCX3CL1 for 24 h. **(H)** Transformation to myofibroblasts assessed by α-SMA (red) staining in NRCF treated with sCX3CL1 or sCX3CL1+CX3CR1 neutralizing antibody or sCX3CL1+LY2157299. Scale bar = 30 μm. Western blotting **(I)** and semi-quantitative analysis **(J)** of E-cadherin, fibronectin, vimentin, and α-SMA expression in NRK-52E cells treated with sCX3CL1 alone or co-treated with either a CX3CR1 neutralizing antibody or LY2157299. **(K)** Representative images of immunostaining for E-cadherin (green), fibronectin (green), vimentin (red), and α-SMA (green) in NRK-52E cells. Scale bar = 30 μm ^*^p < 0.05 *vs*. the sCX3CL1 group. Experiments presented in panel **(B)** was analyzed using two-tailed unpaired *t*-test and in panels **(E)**, **(F)**, and **(J)** were analyzed using one-way ANOVA followed by Bonferroni’s *post hoc* test.

In cultured NRCF and NRK-52E cells, sCX3CL1 treatment increased the expression of TGF-β protein (Figures 3A,B). Exposure to sCX3CL1 also induced phosphorylation of Smad2 and Smad3, as shown by Western blotting (Figures 3C–F). Immunofluorescence also confirmed that sCX3CL1 induced a marked increase of phosphorylated Smad2 and Smad3 in the nucleus of cells (Supplementary Figures S3A–D). Both Western blotting and immunofluorescence demonstrated that exposure to sCX3CL1 induced the fibroblasts into myofibroblasts transition (FMT), which were characterized by the presence of a microfilamentous contractile apparatus enriched with α-smooth muscle actin (α-SMA) (Figures 3G,H). Both Western blotting and immunofluorescence showed a decrease in expression of the epithelial marker E-cadherin by NRK-52E cells in response to sCX3CL1 stimulation, while expression of the mesenchymal markers like Fibronectin, Vimentin, and α-SMA was increased (Figures 3I–K). The above-mentioned effects of sCX3CL1 were blocked by co-treatment with either the CX3CR1 neutralizing antibody (5 μg/ml) or with a TGF-β/Smad inhibitor (LY2157299 at 500 nM) (Figures 3C–K; Supplementary Figures S3A–D), suggesting that sCX3CL1-induced phenotypic trans-differentiation of cardiac and renal cells is dependent on the TGF-β/Smad pathway.

### Inhibition of CX3CL1/CX3CR1 Preserves Cardiorenal Function

**FIGURE 4:**
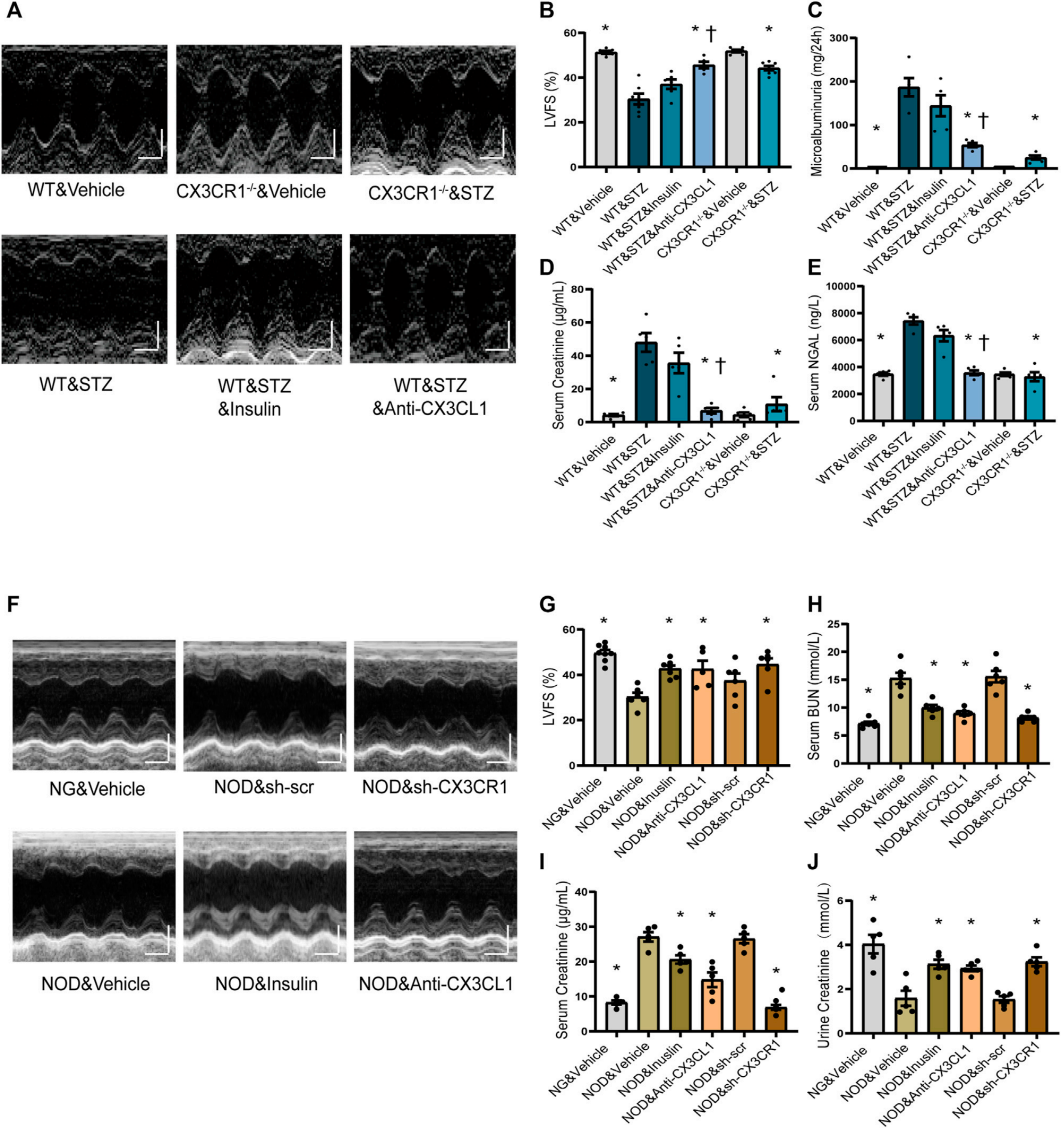
Effect of CX3CL1/CX3CR1 inhibition on cardiorenal dysfunction in mice with diabetes-induced CRS. **(A)** Representative M-mode echocardiographic images of mice at 6 weeks after STZ injection. Scale bars, 2 mm (upper panels), horizontal bars represent 100 ms. **(B)** Left ventricular fractional shortening (LVFS). **(C)** Microalbuminuria amount for 24 h. **(D)** Serum creatinine levels. **(E)** Serum NGAL levels. For panels **(B–E)**, ^*^p < 0.05 *vs*. WT&STZ group; ^†^*p* < 0.05 *vs*. WT&STZ&Insulin group. For panel **(B)**, *n* = 6 in WT&Vehicle, WT&STZ&Inuslin, and CX3CR1^−/−^&Vehicle group; *n* = 7 in WT&STZ and CX3CR1^−/−^&STZ group; *n* = 5 in WT&STZ&anti-CX3CL1 group. *n* = 5 per group in panels **(C–E)**. **(F)** Representative M-mode echocardiographic images. Scale bars, 2 mm (upper panels), horizontal bars represent 100 ms. **(G)** LVFS. **(H)** Concentrations of serum BUN (urea nitrogen). **(I)** Serum creatinine levels. **(J)** Urine creatinine levels. For panel **(G–J)**, ^*^p < 0.05 *vs*. NOD&Vehicle group. In panel **(G)**, *n* = 8 in NG&Vehicle; *n* = 6 in NOD&Vehicle, NOD&sh-scr, and NOD&sh-CX3CR1 group; *n* = 7 in NOD&Inuslin; *n* = 5 in NOD&Anti-CX3CL1. *n* = 6 per group in panel **(H)**; *n* = 5 per group in panels **(I–J)**. Experiments presented in panels **(B–E)** and **(G–J)** were analyzed using one-way ANOVA followed by Bonferroni’s *post hoc* test.

At 6 weeks after induction of DM by injection of STZ, we noted that wild-type (WT) mice developed rough hair and became glassy-eyed with a staggering gait, while these changes were much less marked in CX3CR1^−/−^ STZ-induced diabetic mice (Supplementary Figure S4A; Supplementary video). At 6 weeks, compared to WT&Vehicle group mice, WT diabetic mice had decreased left ventricular fraction shortening (LVFS), left ventricular ejection fraction (LVEF), and increased left ventricular end-systolic diameter (LVEDs), while CX3CR1^−/−^ diabetic mice had improvement of above echo parameters (Figures 4A,B; Supplementary Figures S4D–F). CX3CR1^−/−^ diabetic mice also had lower 24 h urinary albumin excretion (microalbuminuria) (Figure 4C), lower serum levels of creatinine (Figure 4D), and lower serum levels of neutrophil gelatinase-associated lipocalin (NGAL) (Figure 4E). Interestingly, these beneficial effects of CXRCR1 deficiency in STZ-induced diabetic mice were independent of the blood glucose level (Supplementary Figure S4G). Moreover, knockdown of the CX3CR1 results in similar cardiorenal improvement in NOD mice (Supplementary Figures S5A–E, Figures 4F–J). The above results suggest that CX3CR1 deficiency preserves cardiorenal function in diabetic mice.

After hyperglycemia had persisted for 4 weeks, we treated STZ-induced diabetic WT mice and NOD mice with insulin or an CX3CL1 neutralizing antibody for 2 weeks. As a result, the insulin-treated STZ mice remained in a poor condition similar to untreated WT diabetic mice, while the appearance and behavior of mice treated with the CX3CL1 neutralizing antibody showed marked improvement (Supplementary Figure S4A, Supplementary Video). Additionally, in WT diabetic mice, treatment with the CX3CL1 neutralizing antibody, resulted in significant improvement of cardiorenal dysfunction (higher LVFS, lower renal tissue NGAL levels, less microalbuminuria, and lower urine and serum creatinine levels), while insulin treatment only resulted in an improving tendency of cardiorenal dysfunction (Figures 4A–E). In NOD mice, treatment with either CX3CL1 neutralizing antibody or insulin significantly improved cardiorenal dysfunction (Figures 4F–J; Supplementary Figures S5C–E).

### Inhibition of the CX3CL1/CX3CR1 Axis Reduces Apoptosis, Mitochondrial Dysfunction and Fibrosis in Diabetic Mice

**FIGURE 5:**
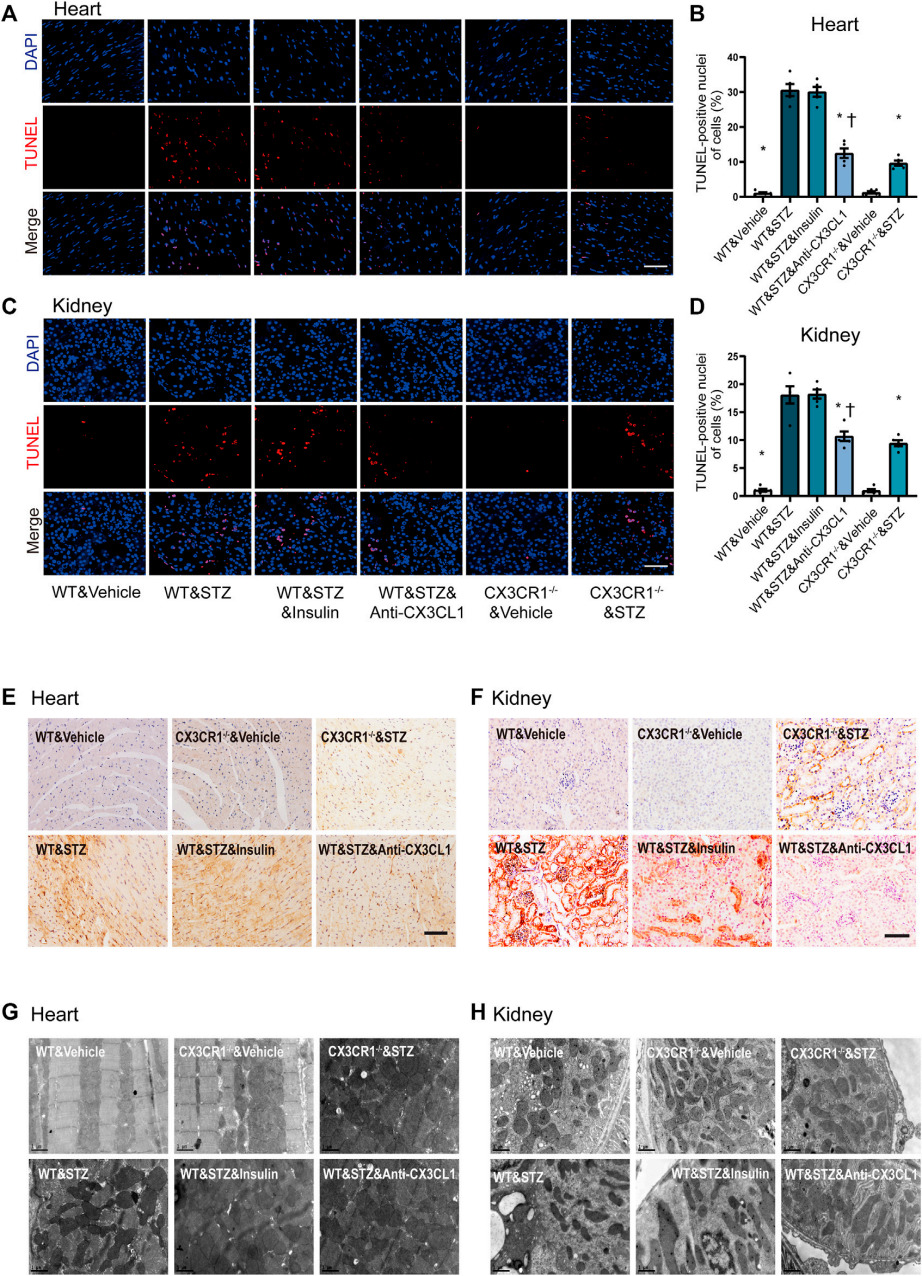
Inhibition of CX3CL1/CX3CR1 attenuated apoptosis in mice with STZ-induced diabetes. **(A)** Representative images of TUNEL staining in heart sections at 6 weeks after STZ-injection. **(B)** Percentage of TUNEL-positive nuclei in heart tissue. **(C)** Representative images of TUNEL staining in kidney sections at 6 weeks after STZ-injection. **(D)** Percentage of TUNEL-positive nuclei in kidney tissue. **(E)** Bax immunostaining (brown staining) in heart tissue. **(F)** Bax immunostaining in kidney tissue. **(G)** Representative electronic micrographs of the heart featuring sarcomeres and mitochondria. **(H)** Representative electronic micrographs of the kidney featuring tubular mitochondria. For panel **(B)** and **(D)**, ^*^p < 0.05 *vs*. WT&STZ mice; ^†^*p* < 0.05 *vs*. WT&STZ&Insulin mice, *n* = 5 in each group. Scale bar = 100 μm in panel **(A)** and **(C)**, Scale bar = 1 μm in panel **(G)** and **(H)**. Experiments presented in panels **(B)** and **(D)** were analyzed using one-way ANOVA followed by Bonferroni’s *post hoc* test.

TUNEL assay revealed an increase of apoptotic cells in both the heart and the kidneys of STZ-induced diabetic WT mice, while fewer apoptotic cells were noted in CX3CR1^−/−^ diabetic mice or WT mice treated with the CX3CL1 neutralizing antibody for 2 weeks (Figures 5A–D). In contrast, insulin treatment did not cause a significant reduction of apoptosis (Figures 5A–D). Immunohistochemical analysis revealed that expression of Bax was enhanced in the heart and kidneys of WT diabetic mice, but not in CX3CR1^−/−^ diabetic mice and WT mice treated with the CX3CL1 neutralizing antibody (Figures 5E,F). The level of Bax expression was similar in WT diabetic mice with or without insulin treatment (Figures 5E,F). Electron microscopy also revealed deformities of some of the myocardial and tubular mitochondria in WT diabetic mice, including attenuation and angulation along the longitudinal axis with focal enlargement and swelling of the cristae (Figures 5G,H). In contrast, the majority of the mitochondria were intact with few broken cristae in CX3CR1^−/−^ diabetic mice or WT diabetic mice treated with the CX3CL1 neutralizing antibody (Figures 5G,H).

### Inhibition of the CX3CL1/CX3CR1 Axis Alleviates Cardiorenal Fibrosis by Restraining Cell Trans-differentiation

**FIGURE 6:**
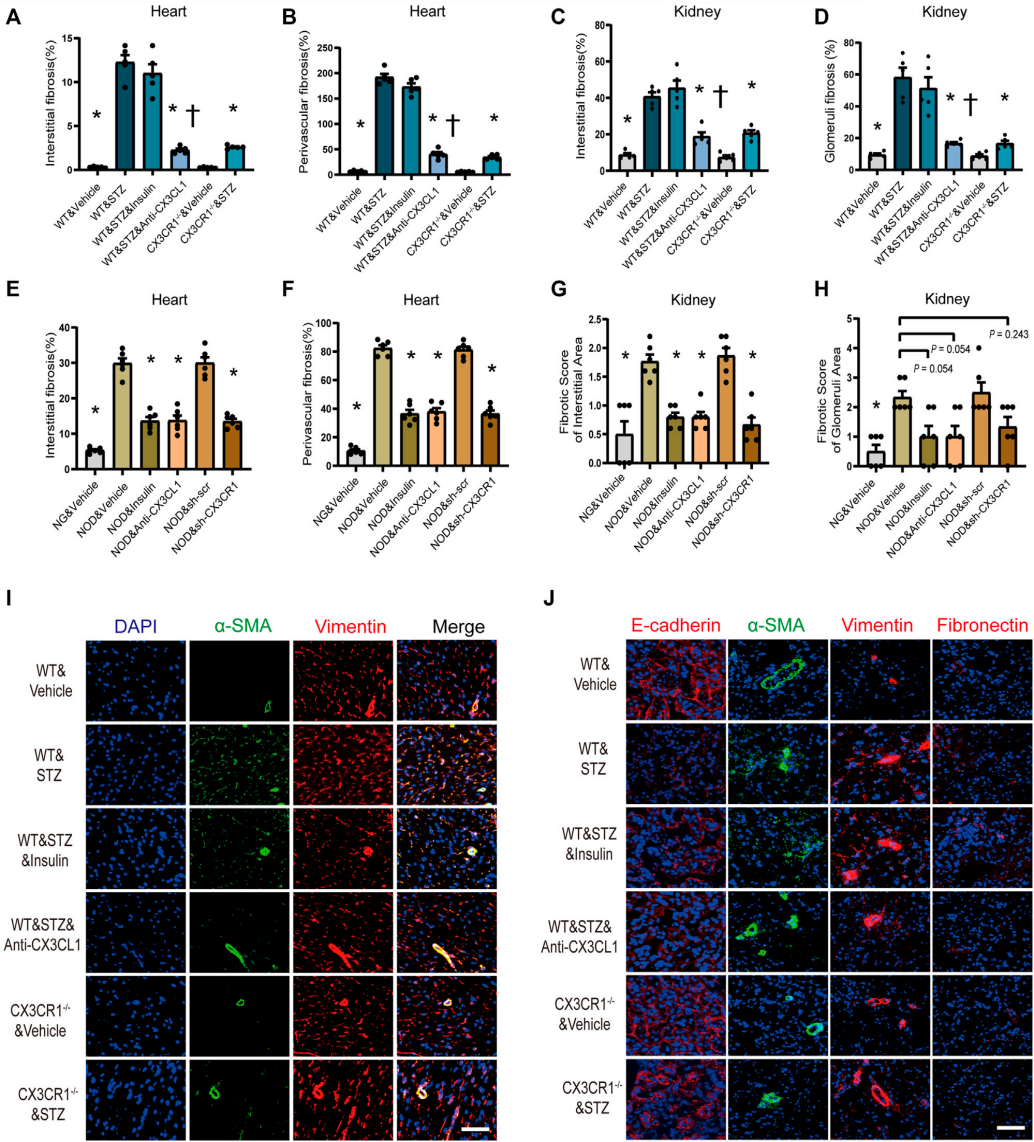
Inhibition of CX3CL1/CX3CR1 attenuated fibrosis in diabetes mice. **(A)** Myocardial interstitial fibrosis in STZ-induced DM mice. **(B)** Perivascular fibrosis of heart in STZ-induced DM mice. **(C)** Fibrotic score of the renal interstitial area in STZ-induced DM mice. **(D)** Fibrotic score of the glomeruli area in STZ-induced DM mice. **(E)** Myocardial interstitial fibrosis in NOD mice. **(F)** Perivascular fibrosis of heart in NOD mice. **(G)** Fibrotic score of the renal interstitial area in NOD mice. **(H)** Fibrotic score of the glomeruli area in NOD mice. **(I)** Representative images of immunostaining for DAPI (blue), α-SMA (green) and vimentin (red) in heart tissue. Scale bar = 100 μm. **(J)** Representative images of immunostaining for E-cadherin (red), fibronectin (red), vimentin (red), and α-SMA (green) in kidney tissue. Scale bar = 100 μm. For panels **(A–D)**, ^*^p < 0.05 *vs*. WT&STZ group; ^†^*p* < 0.05 *vs*. WT&STZ&Insulin mice; *n* = 5 per group. For panels **(E–H)**, ^*^p < 0.05 *vs*. NOD&Vehicle group; *n* = 6 per group. Experiments presented in panels **(A–H)** were analyzed using one-way ANOVA followed by Bonferroni’s *post hoc* test.

Myocardial interstitial and perivascular fibrosis were less prominent in CX3CR1^−/−^ diabetic mice or WT diabetic mice treated with the CX3CL1 neutralizing antibody (Figures 6A,B, Supplementary Figure S6A). When renal fibrosis was assessed by Masson-trichrome staining, there was a significantly lower fibrotic score for renal glomerular and interstitial area in CX3CR1^−/−^ diabetic mice or WT diabetic mice receiving the neutralizing antibody than in untreated WT diabetic mice (Figures 6C,D, Supplementary Figure S6B). Similarly, cardiorenal benefits from knockdown CX3CR1 by AAV or neutralizing antibody improved cardiorenal fibrosis in NOD mice. As for insulin treatment, just in NOD mice, could reverse cardiorenal fibrosis while it did not work in STZ-induced diabetic mice (Figures 6A–H, Supplementary Figures S4A–D).

Immunofluorescence showed that the number of vimentin-positive cardiac fibroblasts with abundant α-SMA protein was markedly increased in STZ-induced diabetic WT mice, indicating that phenomenon of cardiac fibroblasts to myofibroblasts transition (FMT) (Figure 6I). Furthermore, in the kidneys of WT diabetic mice, E-cadherin expression was decreased along with increase of fibronectin, vimentin, and α-SMA expression, suggesting that the epithelial-mesenchymal transition (EMT) had been induced (Figure 6J). In CX3CR1^−/−^ and neutralizing antibody-treated diabetic mice, there was marked suppression of cellular trans-differentiation in the heart and kidneys, but this was not seen in insulin-treated diabetic mice (Figures 6I,J).

### SGLT2i Improves Cardiorenal Dysfunction and Downregulates CX3CL1 Expression in Cardiac and Renal Cells

**FIGURE 7:**
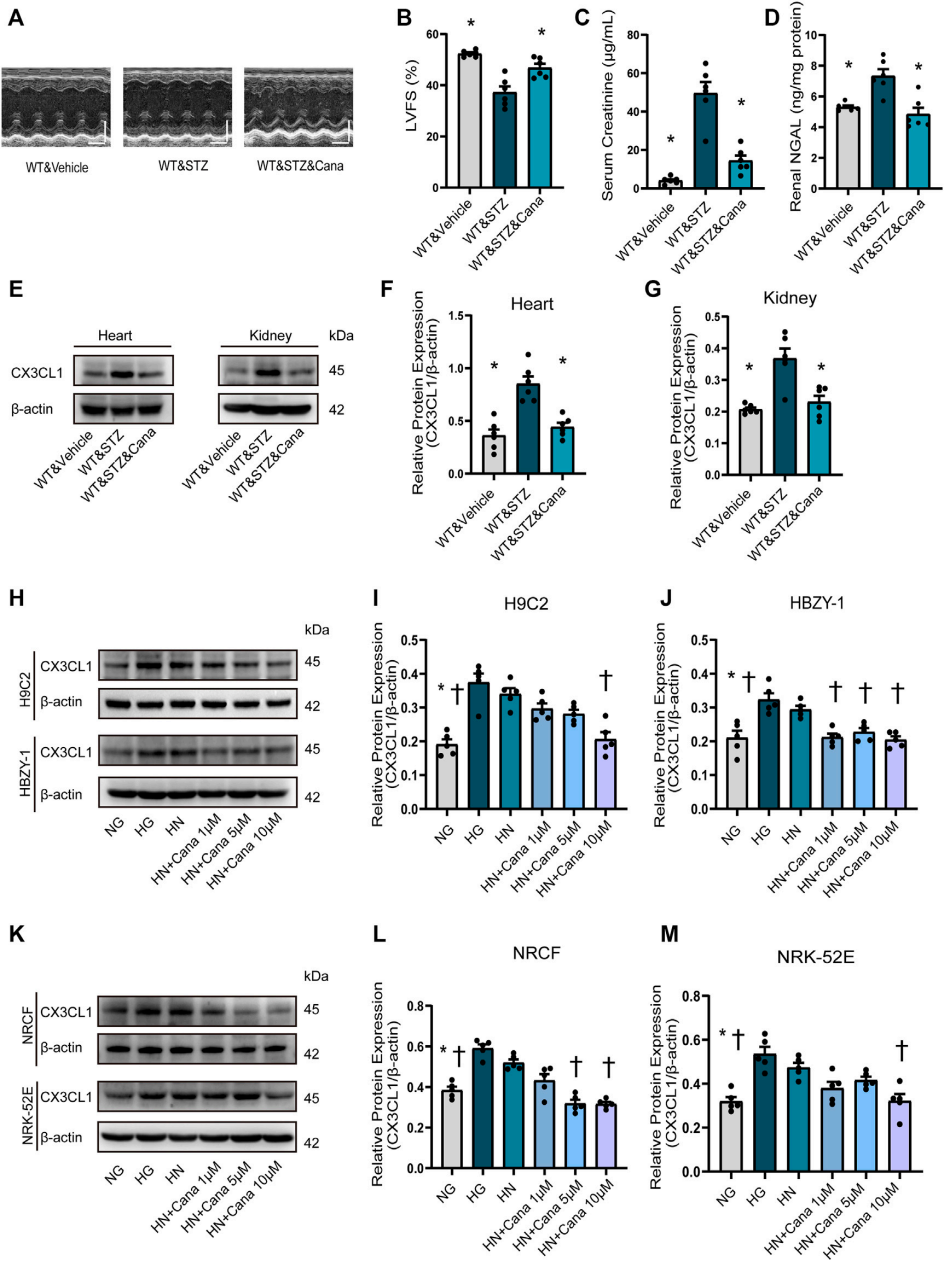
Canagliflozin (Cana) improved cardiorenal dysfunction and repressed diabetic induced or high glucose induced CX3CL1 expression. **(A)** M-mode of echocardiography. Scale bars, 2 mm (upper panels), horizontal bars represent 100 ms. **(B)** Left ventricular fractional shortening (LVFS). **(C)** Serum creatinine concentration. **(D)** Renal neutrophil gelatinase-associated lipocalin (NGAL) content. **(E)** CX3CL1 expression detected by Western blotting in heart and kidney tissues. **(F)** Semi-quantitation of CX3CL1 expression in heart. **(G)** Semi-quantitation of CX3CL1 expression in kidney. ^*^p < 0.05 *vs*. WT&STZ mice; *n* = 6 per group. **(H–M)** Western blotting of CX3CL1 levels in cultured cardiomyocytes (H9C2), glomerular mesangial cells (HBZY-1), neonatal rat cardiac fibroblasts (NRCF) and renal tubular epithelial cells (NRK-52E). The cultured cells were exposed for 5 days either to normal concentration of glucose (5 mM, NG) or high concentration of glucose (33.3 mM, HG) as well as to HG for 3 days followed by NG for 2 days (HN) with/without treatment of Cana. ^*^p < 0.05 *vs*. HG; _†_*p* < 0.05 *vs*. HN; *n* = 5 per group. Experiments presented in panels **(B–D)**, **(F–G)**, **(I–J)**, and **(L–M)** were analyzed using one-way ANOVA followed by Bonferroni’s *post hoc* test.

Finally, we examined if CX3CL1 is a therapeutic target of SGLT2i because it is a consensus that SGLT2i is protective for cardiorenal dysfunction (Rangaswami et al., 2019) and possibly are associated with SGLT2-independent mechanisms. Four weeks after STZ-induced hyperglycemia, we treated the diabetic mice with canagliflozin for 2 weeks. As a result, canagliflozin significantly improved cardiorenal dysfunction (Figures 7A–D), dramatically suppressed the cardiac and renal expression level of CX3CL1 (Figures 7E–G). Considering the glucose-lowering effect of canagliflozin, we tried to test whether canagliflozin had a glucose-lowering independent effect on CX3CL1 expression. Three days after exposure to high glucose (HG, 33 mM), the medium of cardiac and renal cells was replaced with normal glucose medium (HN, 5.5 mM) and cultured for 2 days in the presence/absence of canagliflozin. As shown in Figures 7H–M, there was still a high level of CX3CL1 expression in the HN groups, while co-treatment with canagliflozin 10 μM significantly downregulated the protein expression of CX3CL1 in the four types of cardiac and renal cells.

## Discussion

The results of the present study support an important role of CX3CL1 in DM-induced CRS5 and CX3CL1 would be a new therapeutic target of cardiorenal dysfunction. Our findings demonstrated that: (1) CX3CL1 levels are significantly increased in four types of cardiac and renal cells by exposure to high glucose; (2) The proapoptotic and profibrotic effects of CX3CL1 are medicated by the RhoA/ROCK1-Bax and TGF-β/Smad signaling pathways, respectively; (3) Inhibition of the CX3CL1/CX3CR1 axis preserves cardiorenal function in diabetic mice by preventing apoptosis, mitochondrial dysfunction, and cellular transition like FMT and EMT; (4) SGLT2i markedly suppresses the diabetic-induced or HG-induced cardiorenal CX3CL1 expression.

**FIGURE 8:**
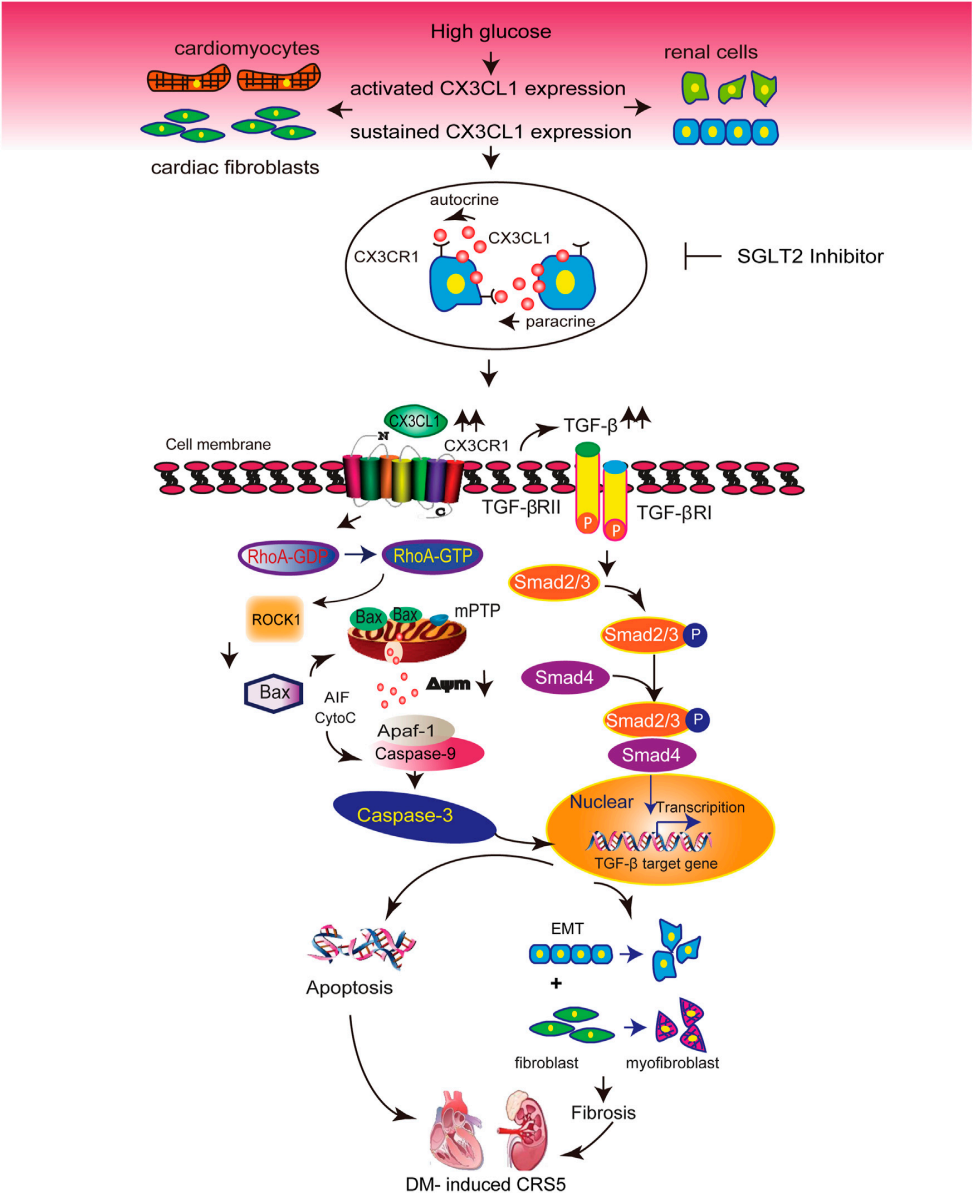
Illustration of the molecular mechanisms by which CX3CL1 promotes cardiorenal dysfunction induced by diabetics. CX3CL1 expression is upregulated in cardiac and renal cells by a high glucose environment. Persistent high CX3CL1 expression accelerates the mitochondrial apoptotic pathway through activation of RhoA/ROCK1-Bax signaling. In addition, CX3CL1 regulates fibroblast and epithelial cell phenotypic trans-differentiation through activation of TGF-β/Smad signaling. CX3CL1 leads to the onset of cardiorenal dysfunction in diabetes-induced cardiorenal syndrome type 5 (CRS5) due to its proapoptotic and profibrotic effects, while SGLT2 inhibitor could improve CRS5 at least partially by repressing CX3CL1 expression.

Diabetic cardiomyopathy and nephropathy are common and serious complications of DM (Li et al., 2012; Satirapoj, 2012). Thus, heart failure and renal dysfunction frequently co-exist in patients with DM(Karnib and Ziyadeh, 2010), and these conditions may share some common mechanisms. Our *in vitro* and *in vivo* studies demonstrated that CX3CL1 expression was significantly increased in cardiac and renal cells exposed to diabetic conditions. These findings, combined with previous work from our group and others on the deleterious effects of CX3CL1 in cardiovascular and renal disease (Oh et al., 2008; Koziolek et al., 2010; Xuan et al., 2011; Xuan et al., 2012; Song et al., 2013), suggest that CX3CL1 may be involved in the development of cardiorenal dysfunction in diabetes. We and others have demonstrated cultured cardiac and renal cells express CX3CR1. Thus, the increased CX3CL1 induced by high glucose may directly interact with its receptor (CX3CR1) via autocrine or paracrine manner, just as shown in Figure 8.

In the present study, we verified the occurrence of this phenomenon in 2 mouse models of type 1 DM. As to STZ-induced diabetic mice, we observed that achieving glycemic control with insulin therapy at a late stage failed to prevent the progression of cardiorenal dysfunction and that upregulation of CX3CL1 in the heart and kidneys did not respond to improved glycemic control. Interestingly, NOD mice got cardiorenal benefits from CX3CL1 neutralizing antibody or AAV-mediated CX3CR1 knockdown and insulin treatment. In patients with DM, insulin is neutral on cardiovascular outcome or even promotes cardiac remodeling (The BARI 2D Study Group et al., 2009; The ORIGIN Trial Investigators et al., 2012). In NOD mice, it is also controversial whether insulin therapy could improve the progression and prognosis of diabetes (Muir et al., 1995; Karounos et al., 1997; Grönholm et al., 2017). The inconsistent results on the effects of insulin in STZ-induced DM and NOD mice may be related to variance between environmental differences and biological variation (Reed and Herold, 2015).

It has been demonstrated that apoptosis, fibrosis, and mitochondrial dysfunction contribute to the development of diabetic cardiomyopathy and nephropathy (Lin et al., 2006; Menini et al., 2006; Kuethe et al., 2007; Sun et al., 2008; Ti et al., 2011). It has been reported that cleavage of ROCK1 and RhoA/ROCK1-Bax signaling promote apoptosis associated with myocardial hypertrophy and heart failure (Chang et al., 2006; Del Re et al., 2007; Shi et al., 2010). Consistent with these reports, we found that sCX3CL1 directly activated RhoA/ROCK1-Bax signaling, which led to mitochondrial dysfunction and apoptosis in cultured cardiomyocytes and renal mesangial cells. The occurrence of phenotypic trans-differentiation and its profibrotic effect have been confirmed in both cardiac and renal remodeling (Dobaczewski et al., 2010; Liu, 2010). We showed that CX3CL1 induced FMT or EMT in a TGF-β/Smad-dependent manner. Although previous studies have demonstrated that CX3CL1 is involved in cardiac and renal fibrosis (Koziolek et al., 2010; Xuan et al., 2011), our current findings have identified a novel CX3CL1 signaling pathway related to the progression of fibrosis. We also demonstrated that CX3CR1 deficiency simultaneously ameliorated apoptosis, mitochondrial dysfunction, and fibrosis in diabetic hearts and kidneys, thus leading to improvement of cardiorenal dysfunction and survival. These results suggest that the CX3CL1/CX3CR1 system has a critical role in the diabetic CRS type 5.

It was reported that the CX3CL1/CX3CR1 system plays a beneficial role in the maintenance of appropriate insulin secretion and glycemic control (Lee et al., 2013), suggesting that CX3CL1 is a versatile factor which exerts pleiotropic effects in DM. Since β-cell secretory function is severely impaired in STZ-induced models of type 1 diabetes (Tourrel et al., 2001), the effect of CX3CL1 on insulin secretion would be difficult to identify in the present study. In particular, we found that the improvement of cardiorenal dysfunction in diabetic CX3CR1-deficient mice or in diabetic WT mice receiving CX3CL1 neutralizing antibody treatment was independent of changes in the blood glucose level, implying that enhancement of insulin secretion by CX3CL1 may be insufficient to stop the progression of CRS type 5. More evidence supports that initial increase of insulin secretion by CX3CL1/CX3CR1 may be just a defensive reaction to inflammation, which can lead to chronic hyperinsulinemia and depletion of functional reserves of β-cells (Bergmann and Sypniewska, 2014).

Circulating levels of sCX3CL1 are increased in patients with type 2 DM and in patients with heart failure (Husberg et al., 2008; Escher et al., 2011; Shah et al., 2011; Richter et al., 2012). Enhanced cardiac and renal CX3CL1 expression in the diabetic state might result in an increase of circulating or local sCX3CL1. It is widely accepted that the integrity of the endothelial barrier is highly dependent on actin cytoskeleton-mediated adherent junctions (Aslam et al., 2011). Previous study demonstrated CX3CL1 induced F-actin rearrangement in endothelial cells (Volin et al., 2010), which indicates that CX3CL1 may be involved in the regulation of endothelial permeability. Thus, the increased local sCX3CL1 might cross the endothelial barrier, which partly contributes to the increased circulating sCX3CL1 in diabetes. Except kidney and heart, there are many tissues in diabetes that express CX3CL1 such as adipose, brain, and so on (Shah et al., 2011; Kawamura et al., 2021). It is possible that the above tissues release soluble CX3CL1 into the circulatory system. To explore the changing of circulating CX3CL1 with cardiac/renal CX3CL1 is a complicated but valuable topic. Taken together, it seems that CX3CL1/CX3CR1 system may play an important role in cardiorenal crosstalk via circulating sCX3CL1 in diabetes. Although it has already been demonstrated that CX3CL1 exacerbates cardiac and renal dysfunction in several animal models (Koziolek et al., 2010; Xuan et al., 2011), in this study we provided the further evidence that CX3CL1 has a role in both cardiac and renal dysfunction in two diabetic mouse models. Therefore, the pathophysiological role of CX3CL1 in cardiorenal cross-talk should be explored in other types of CRS.

Most of the glucose-lowing agents are neutral for cardiorenal protection, and some of them are detrimental for heart failure. Although CX3CL1 was reported to increase insulin secretion, it would be doubtful to use CX3CL1 as a glucose-lowing agent due to its detrimental effect on cardiorenal function. SGLT2i is strongly recommended for the treatment of diabetic patients because of its significant cardiorenal benefits. It was reported that there was no or very low expression of SGLT2 in the heart (Jiang et al., 2021), but SGLT2i improves heart failure and chronic kidney disease in patients with/without DM (Martinez et al., 2020; Wheeler et al., 2021), suggesting SGLT2-independent and glucose-lowering independent effects of SGLT2i. In fact, it is far from clear for the mechanisms of SGLT2i improving cardiorenal pathophysiology. In this study, we confirmed that canagliflozin significantly improved diabetes-induced cardiorenal dysfunction, and we also provided first evidence that diabetic or high glucose-induced CX3CL1 expression was inhibited markedly by canagliflozin in a glucose-lowering independent manner, suggesting that CX3CL1 is a potential target of SGLT2i. Recently, Nami Lee et al, (2021) reported that SGLT2i exerts potential anti-inflammatory functions by attenuating the secretion and mRNA expression of proinflammatory cytokines, such as TNF-α, IL-1β, and IFN-γ, which may, as soluble mediators, affect the action of CX3CL1 (Von Vietinghoff and Kurts, 2021). It has been reported that p53, a transcript factor of CX3CL1 (Shiraishi et al., 2000), is elevated in diabetic cardiomyopathy and nephropathy (Gu et al., 2018; Ma et al., 2020). Moreover, SGLT2i treatment has an inhibiting effect on p53 (Park et al., 2020), which indicates that p53 might be a potential mediator between SGLT2i and CX3CL1. Future jobs are needed to clarify whether and how SGLT2i improves cardiorenal dysfunction induced by activation of CX3CL1/CX3CR1.

Our findings revealed that persistent expression of CX3CL1 and its proapoptotic and profibrotic effects directly contribute to the progression of CRS5 in diabetic mice. Accordingly, inhibition of the CX3CL1/CX3CR1 system by neutralizing antibody of CX3CL1 or silencing of CX3CR1 or SGLT2i may represent a novel therapeutic strategy for improving cardiorenal dysfunction in diabetes.

## Data Availability

The original contributions presented in the study are included in the article/Supplementary Materials, further inquiries can be directed to the corresponding authors.

## Abbreviations

CRS5, cardiorenal syndrome type 5; DM, diabetes mellitus; NGAL, neutrophil gelatinase-associated lipocalin; NOD, non-obese diabetic; STZ, streptozotocin; WT, wildtype.
