# Supplementary material for: CX3CL1 Worsens Cardiorenal Dysfunction and Serves as a Therapeutic Target of Canagliflozin for Cardiorenal Syndrome
Source: Front Pharmacol. 2022 Mar 18;13:848310. doi: 10.3389/fphar.2022.848310 (PMC8971671; doi:10.3389/fphar.2022.848310)
Supplement: Supplementary file 1 [file Table1.DOCX]

**Supplementary Information**

**CX3CL1 Worsens Cardiorenal Dysfunction and Serves as A Therapeutic Target of Canagliflozin for Cardiorenal Syndrome**

Cankun Zheng^1^, Wanling Xuan^1^* Zhenhuan Chen^1,2^, Rui Zhang^1^, Xiaoxia Huang^1^, Yingqi Zhu^1^, Siyuan Ma^1^, Kaitong Chen^1^, Lu Chen^1^, Mingyuan He^1^, Hairuo Lin^1^, Wangjun Liao^3^, Jianping Bin^1,4^, Yulin Liao^1,4^*

^1^ Department of Cardiology, State Key Laboratory of Organ Failure Research, Guangdong Provincial Key Laboratory of Shock and Microcirculation, Nanfang Hospital, Southern Medical University, Guangzhou 510515, China

^2^ Department of Cardiology, Jiangxi Provincial People's Hospital Affiliated to Nanchang University, Nanchang, Jiangxi, 330006, China

^3^ Department of Oncology, Nanfang Hospital, Southern Medical University, Guangzhou Guangdong 510515, China

^4^ National Clinical Research Center of Kidney Disease, Guangdong Provincial Institute of Nephrology, Nanfang Hospital, Southern Medical University, Guangzhou, 510515, China.

*Address correspondence to:

Yulin Liao, Department of Cardiology, Nanfang Hospital, Southern Medical University, 1838 Guangzhou Avenue north, Guangzhou, 510515, China. Phone: 86-20-62786294; Fax: 86-20-87277521; E-mail: [Liao18@msn.com](mailto:Liao18@msn.com) or to Dr. Wanling Xuan with e-mail: [xuanwanling@gmail.com](mailto:xuanwanling@gmail.com).

**Running Title:** CX3CL1 Worsens Cardiorenal Dysfunction

**Table S1. Sequences of primers for RT-PCR**

| **Transcripts** | **Sequence** | |
| --- | --- | --- |
| β-actin（rat） | (F) 5’- GCAGGAGTACGATGAGTCCG -3’ |  |
|  | (R) 5’- ACGCAGCTCAGTAACAGTCC -3’ |  |
| CX3CL1（rat） | (F) 5’-CCTCGGCATGACGAAATGCA-3’ |  |
|  | (R) 5’-AGGCCCTGGAGATTTCTCTG-3’ |  |
| CX3CR1（rat） | (F) 5’-TCCCGGAATTGGATCTAGAG-3’  (R) 5’-GCAGGACCTCGGGGTAATCA-3’ |  |
| β-actin（mouse） | (F) 5’-GTACCACCATGTACCCAGGC-3’  (R) 5’-AACGCAGCTCAGTAACAGTCC-3’ |  |
| CX3CL1（mouse） | (F) 5’-ACGAAATGCGAAATCATGTGC-3’  (R) 5’-CTGTGTCGTCTCCAGGACAA-3’ |  |
| CX3CR1（mouse） | (F) 5’-GAGTATGACGATTCTGCTGAGG-3’  (R) 5’-CAGACCGAACGTGAAGACGAG-3’ |  |

**Table S2. Antibodies used in this study.**

| Antibodies | Species | Dilution | Company | Catalogue |
| --- | --- | --- | --- | --- |
| CX3CL1 | Rabbit | 1:1000 for WB, 1:200 for IHC | Abcam | ab25088 |
| Bax | Mouse | 1:1000 for WB, 1:200 for IHC | Santa | sc-7480 |
| Cytochrome C | Mouse | 1:1000 for WB, 1:500 for IF | Abcam | ab110325 |
| AIF | Rabbit | 1:1000 for WB, 1:200 for IF | CST | 5318s |
| β-actin | Rabbit | 1:1000 for WB | CST | 4970s |
| Smad2/Smad3 | Mouse | 1:1000 for WB | CST | 8685s |
| phospho-Smad2 | Rabbit | 1:1000 for WB, 1:400 for IF | CST | 18338s |
| Phospho-Smad3 | Rabbit | 1:1000 for WB, 1:200 for IF | CST | 8769s |
| TGF-β | Rabbit | 1:1000 for WB | CST | 3711s |
| α-SMA | Rabbit | 1:5000 for WB, 1: 500 for IF | Bimake | A5550 |
| Vimentin | Rabbit | 1:1000 for WB, 1:200 for IF | CST | 5741S |
| E-cadherin | Mouse | 1:1000 for WB, 1:200 for IF | CST | 14472s |
| Fibronectin | Rabbit | 1:1000 for WB, 1:200 for IF | Abcam | ab45688 |

WB: western blot; IHC: immunohistochemistry; IF: immunofluorescence


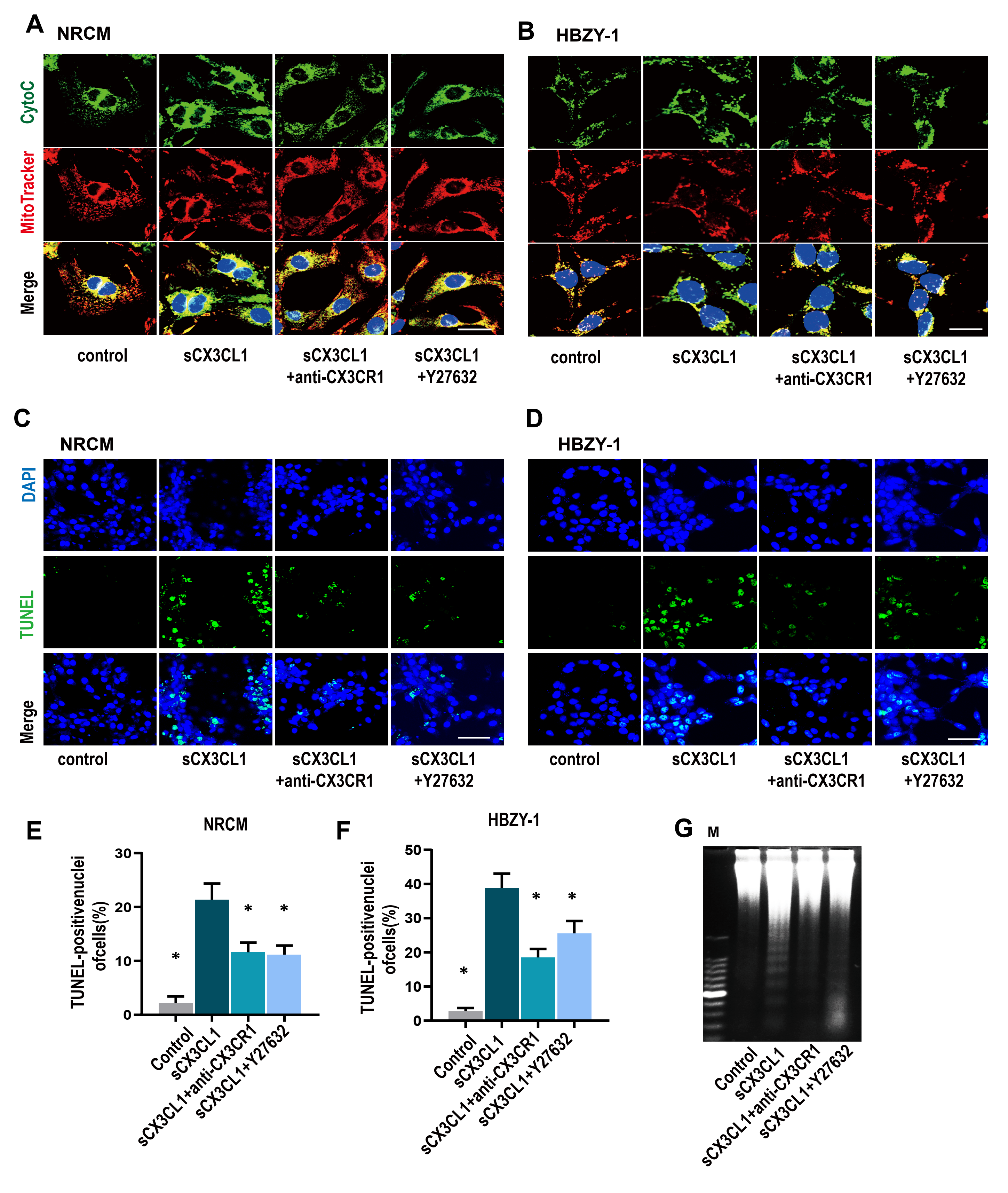


**Figure S1. sCX3CL1 promotes apoptosis in cardiomyocytes and renal cells.** Subcellular localization of cytochrome C (CytoC) was detected in neonatal rat cardiomyocytes (NRCM) **(a)** and HBZY-1 **(b)** after soluble CX3CL1 (sCX3CL1) stimulation or co-treatment with either a CX3CR1 (CX3CL1 receptor) neutralizing antibody or Y-27632 (an inhibitor of ROCK). Scale bar=30 μm. SCX3CL1 induced an increase of apoptosis in NRCM **(c)** and HBZY-1 **(d)** detected by the TUNEL assay. Scale bar=50 μm. The percentage of TUNEL-positive nuclei was calculated in NRCM **(e)** and HBZY-1 **(f)**. **(g)** Representative image of DNA ladders in HBZY-1 cells. ^*^*P*<0.05 vs. sCX3CL1 group, n=5 in each group, Mean±SEM. sCX3CL1


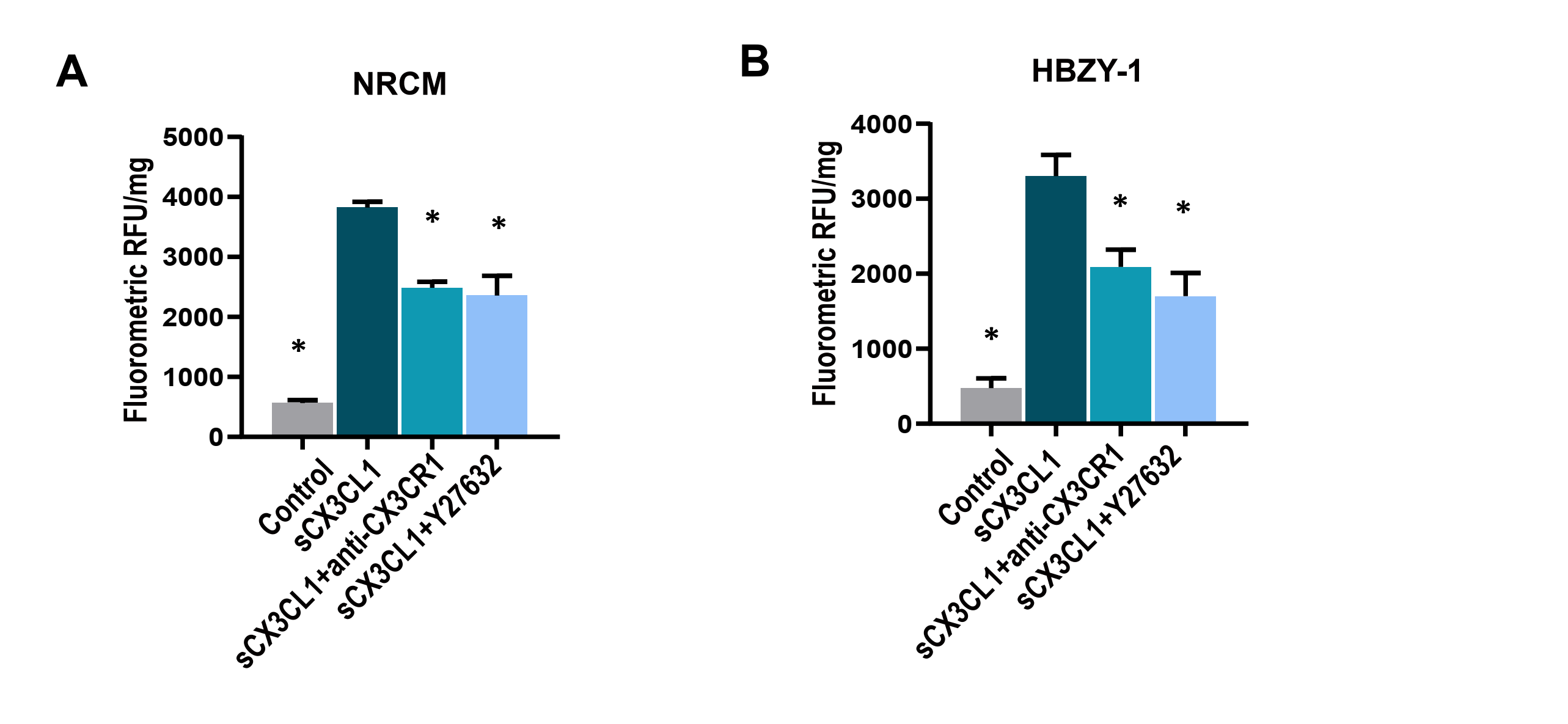


**Figure S2. sCX3CL1 activates caspase-9 in cardiomyocytes and renal cells.** Activity of caspase-9 in neonatal rat cardiomyocytes (NRCM) **(a)** and HBZY-1 **(b)** exposed to soluble CX3CL1 (sCX3CL1) 200 ng/mL in the presence of neutralizing antibody of CX3CR1 5 μg/mL (CX3CL1 receptor) or Y27632 (an inhibitor of ROCK) 10 μM. ^*^*P*<0.05 vs.sCX3CL1. Mean±SEM, experiments were repeated 3 times.


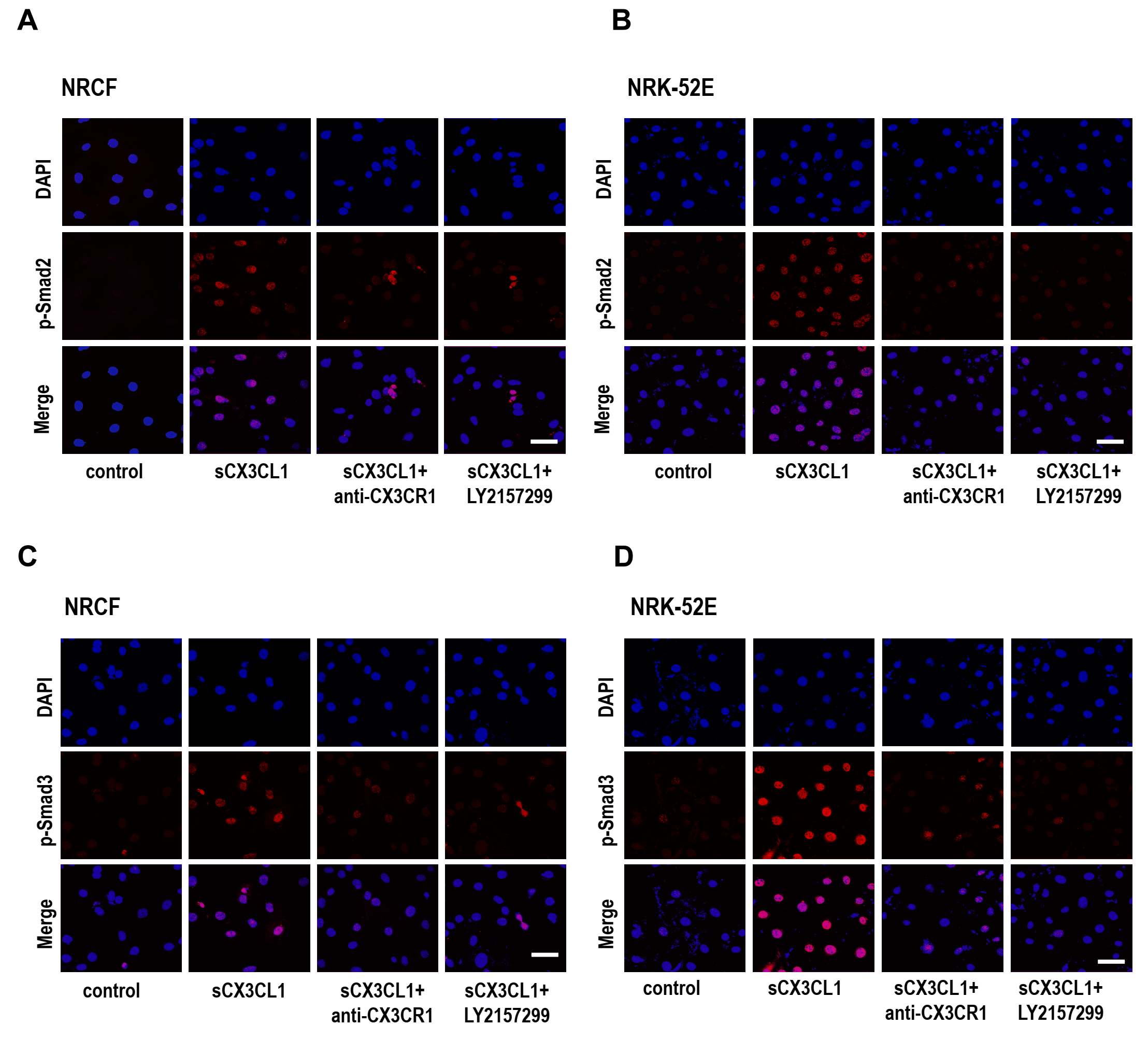


**Figure S3. sCX3CL1 promotes translocation of phosphorylation of Smad2 and Samd3 in cardiac and renal cells.** Subcellular localization analysis of phosphorylation of Smad2 in neonatal rat cardiac fibroblasts (NRCF) **(a)** and NRK-52E **(b)** after exposure to sCX3CL1 stimulation alone or with co-treatment by either a CX3CR1 neutralizing antibody or a TGF-β/Smad inhibitor (LY2157299). Subcellular localization analysis of phosphorylation of Smad3 in NRCF **(c)** and NRK-52E **(d)**. Scale bar=50 μm.


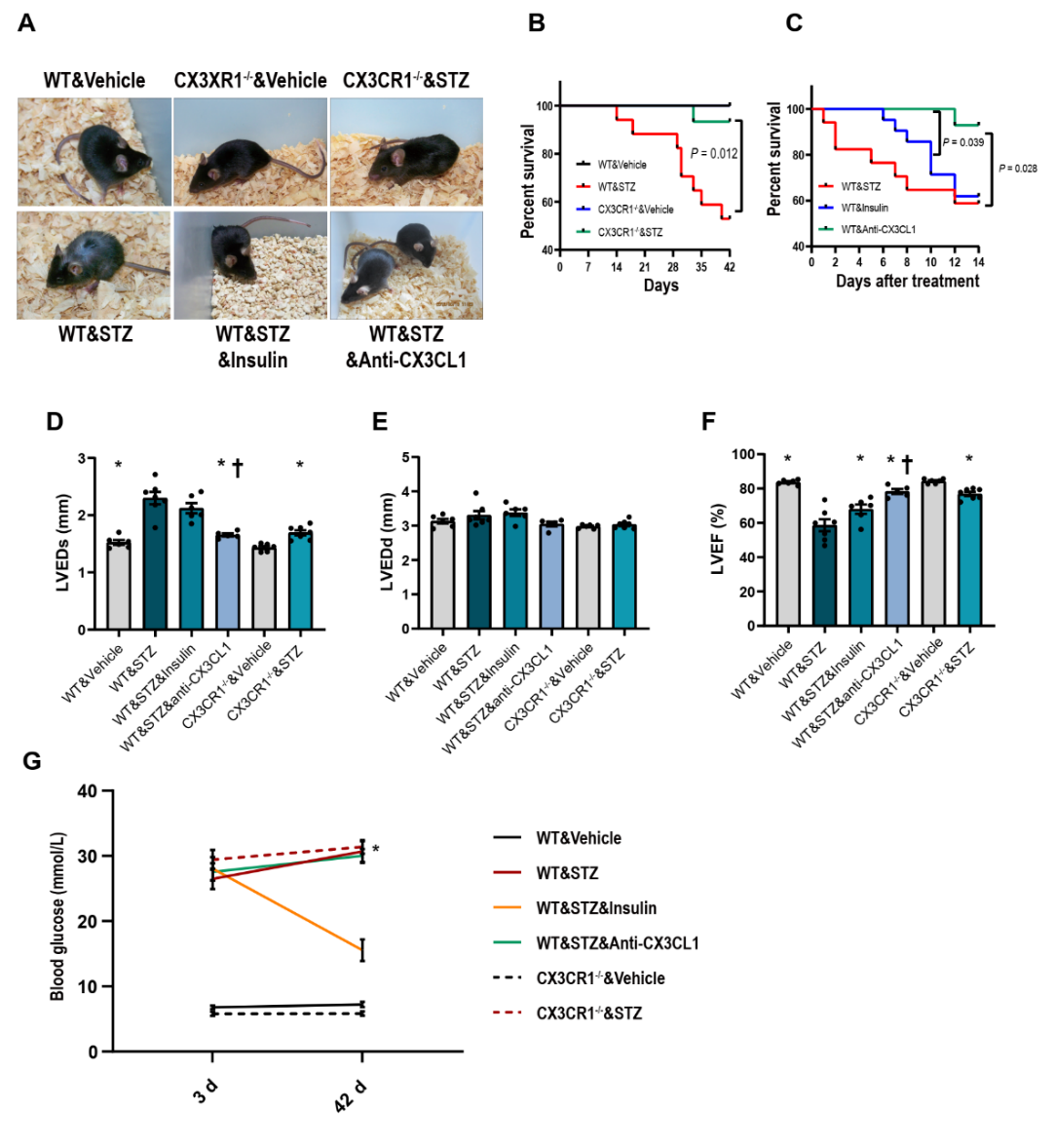
**Figure S4. Inhibition of CX3CL1/CX3CR1 improved the survival rate and cardiac function of mice with STZ-induced diabetes that was independent of blood glucose concentration. (a)** Behavior and appearance of mice with streptozotocin (STZ)-induced diabetes mellitus. **(b)** Kaplan-Meier survival curves for wild-type (WT)&Vehicle mice (n=7), CX3CR1^-/-^&Vehicle mice (n=7), WT&STZ mice (n=17), and CX3CR1^-/-^ &STZ mice (n=15). WT&STZ mice vs. CX3CR1^-/-^ &DM mice, *P*=0.012. **(c)** Kaplan-Meier survival curves for WT&STZ mice (n=17) and WT&STZ mice after treatment with insulin (n=21) or an anti-CX3CL1 neutralizing antibody (n=14) for 14 days. WT&STZ mice vs. WT&STZ&Insulin mice, *P*=0.039; WT DM mice vs. WT&STZ&Anti-CX3CL1 mice, *P*=0.028. **(D)** Left ventricular end-systolic diameter (LVEDs). **(E)** Left ventricular end-diastolic diameter (LVEDd). **(F)** Left ventricular ejection fraction (LVEF). For panel (D) and (F), ^*^*P*<0.05 vs. WT&STZ group; ^†^*P*<0.05 vs. WT&STZ&Insulin group. For panel (D-F), n=6 in WT&Vehicle， WT&STZ&Inuslin, and CX3CR1^-/-^&Vehicle group; n=7 in WT&STZ and CX3CR1^-/-^&STZ group; n=5 in WT&STZ&anti-CX3CL1 group. **(G)** Blood glucose concentrations at 3 and 42 days. Insulin (40 IU/kg/d) and anti-CX3CL1 (neutralizing antibody of CX3CL1, 40 mg/kg/d) were initiated at the 4th week and persisted for 2 weeks. ^*^*P*<0.01 vs. WT&STZ&Insulin group, n = 8 per group.


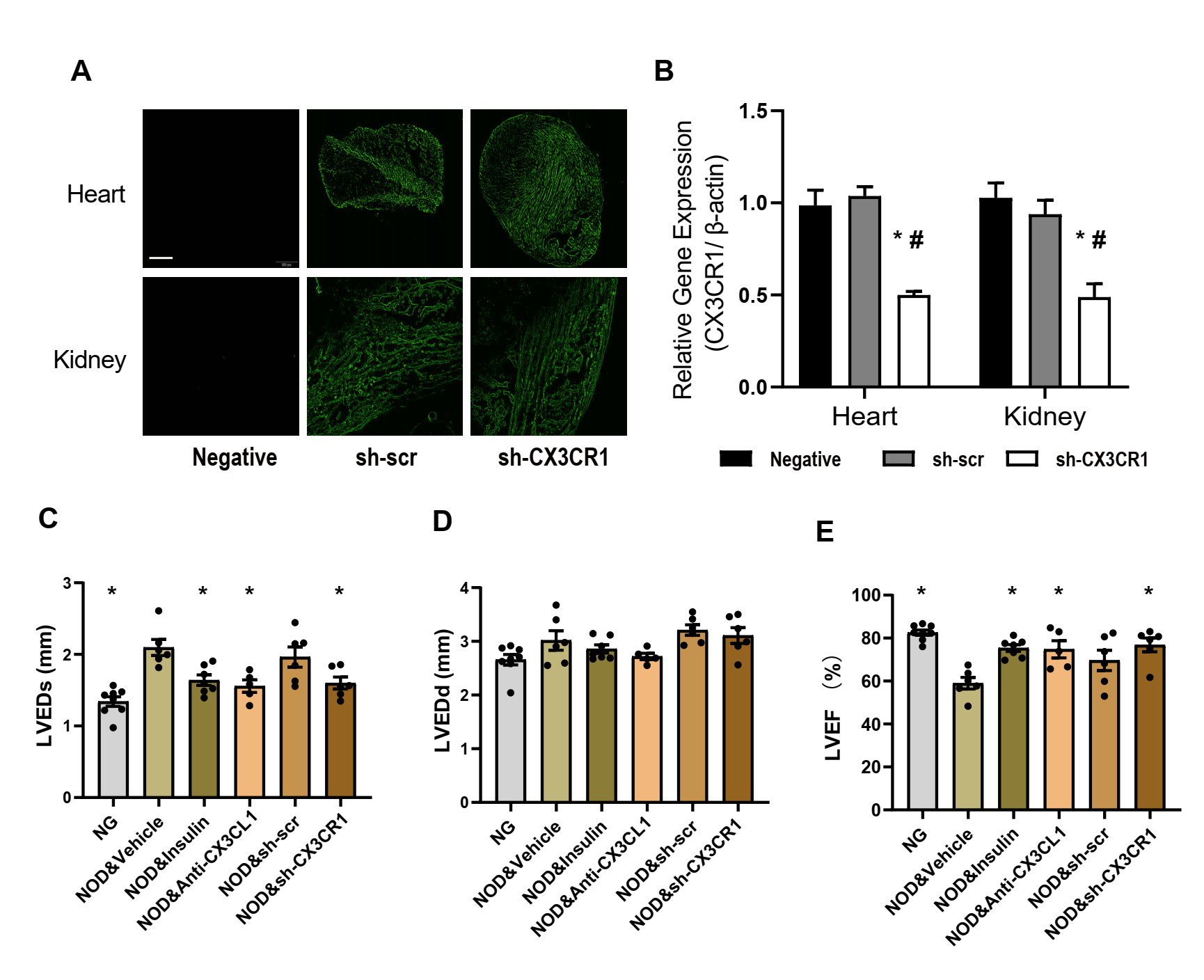


**Figure S5. Verification of adeno-associated virus serotype 9 (AAV9) carrying the short hairpin-CX3CR1 plasmid (sh-CX3CR1) or a scramble plasmid (sh-scr) into the myocardium or kidneys and its effect on cardiac function of NOD mice. (a)** Representative images showing AAV9 infection by fluorescence detection. Scale bar=200 μm. **(b)** Real-time PCR for cardiac and renal CX3CR1 expression levels after silencing. ^*^*P*<0.05, vs. Negative; ^#^*P*<0.05, vs. sh-scr. n=5 in each group. Mean±SEM. **(C)** Left ventricular end-systolic diameter (LVEDs). **(D)** Left ventricular end-diastolic diameter (LVEDd). **(E)** Left ventricular ejection fraction (LVEF). For panel (C-E), n=8 in NG&Vehicle; n=6 in NOD&Vehicle, NOD&sh-scr, and NOD&sh-CX3CR1 group; n=7 in NOD&Inuslin; n=5 in NOD&Anti-CX3CL1. For panel (D) and (E), ^*^*P*<0.05 vs. NOD&Vehicle group.


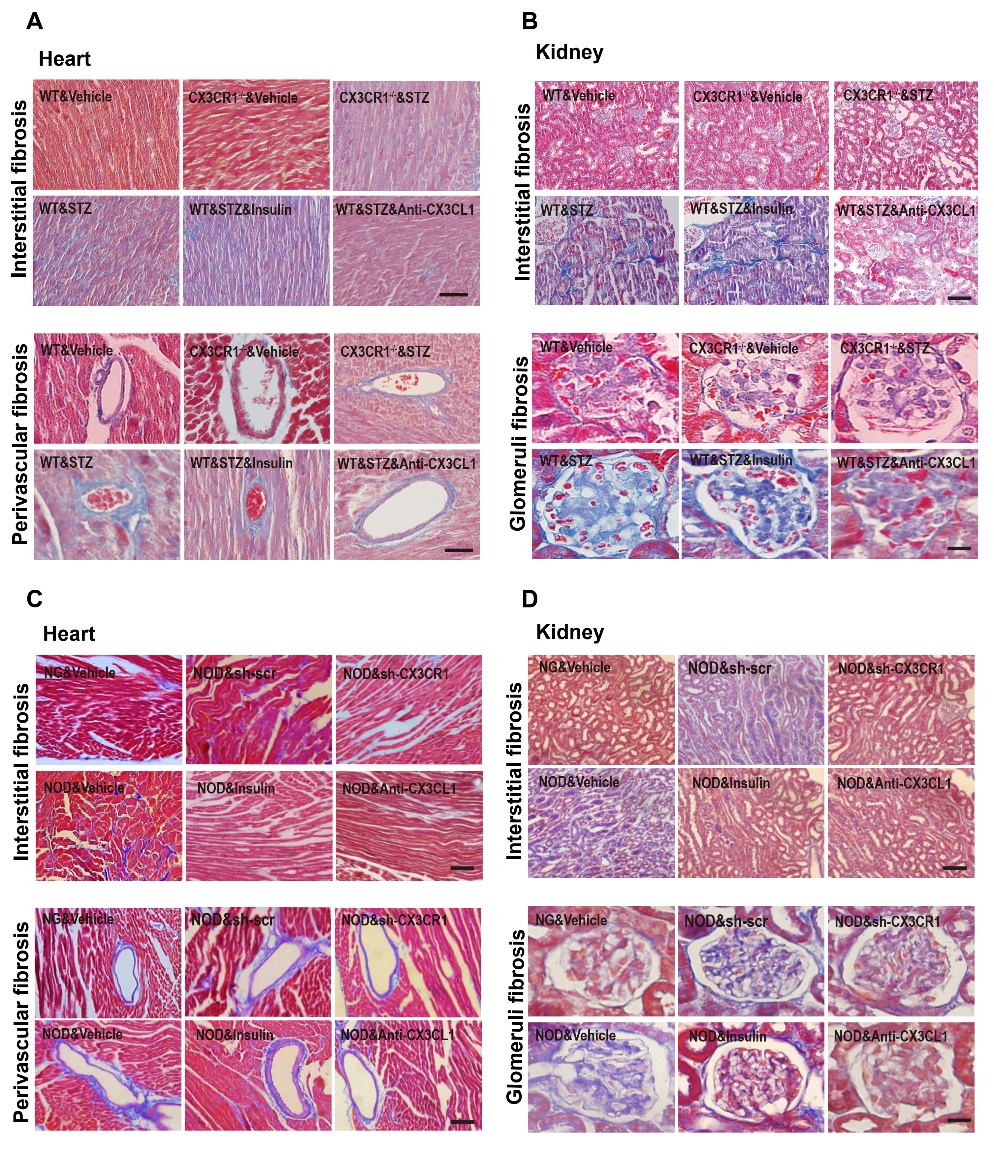


**Figure S6. Inhibition of CX3CL1/CX3CR1 attenuated Cardiorenal fibrosis in mice with diabetes.** Representative Masson’s trichrome staining of heart **(a)** and kidney **(b)** tissues from STZ-induced DM mice, heart **(c)** and kidney **(d)** from NOD mice. Scale bar=100 μm.

**Supplemental Movie Legends**

At 6 weeks after induction of diabetes mellitus (DM) by injection of STZ, wild-type (WT) mice developed rough hair and became glassy-eyed with a staggering gait, while these changes were much less marked in CX3CR1^-/-^ mice. Furthermore, the insulin-treated mice remained in a poor condition similar to untreated WT diabetic mice, while the appearance and behavior of mice treated with the CX3CL1 neutralizing antibody showed marked improvement. WT, wildtype; STZ, streptozotocin.
